# Supplementary material for: Human biomass movement exceeds the biomass movement of all land animals combined
Source: Nat Ecol Evol. 2025 Oct 27;9(12):2259–64. doi: 10.1038/s41559-025-02863-9 (PMC12680537; doi:10.1038/s41559-025-02863-9)
Supplement: Supplementary file 1 — Supplementary Figs. 1–6, Discussion and Tables 1–14. [file 41559_2025_2863_MOESM1_ESM.pdf]

nature ecology & evolution

Supplementary information https://doi.org/10.1038/s41559-025-02863-9

Human biomass movement exceeds the biomass movement of all land animals combined

In the format provided by the authors and unedited

Table of contents

HUMANS 2
WILD TERRESTRIAL MAMMALS EXCLUDING BATS 10
WILD BIRDS 13
TERRESTRIAL ARTHROPODS 18
UPPER BOUND ESTIMATES FOR OTHER TERRESTRIAL ANIMALS 21
DOMESTICATED ANIMALS 23
MARINE ANIMALS 24
SENSITIVITY AND UNCERTAINTY ANALYSIS 28
PASSIVE ANIMAL MOVEMENT 42
MIGRATIONS, GATHERINGS AND COMMUTES 43
ENERGY ESTIMATES 45
REFERENCES 46

Humans

The total biomass of humans is estimated at ≈430 Mt[9], based on a global population of ≈8 billion people and an average biomass of ≈54 kg per person[9]. Databases of road and rail mobility[38, 39,232] report country-level data of human mobility in units of passenger-kilometer per year (pkm/yr) and per mobility mode. Preference was always given to the latest year available. Data compilation was partially drawn from[40] for road and rail transport. Here, this data collection was refined and updated to cover more countries and recent years, as well as walking and cycling mobility and freight transport[39,41–63,232], as detailed in file S1. We also extrapolated the estimates to countries that lacked data and estimated uncertainty bounds (see next paragraph). In total, we extracted road traffic and road freight data for 50 countries each, rail data for 100 countries, walking data for 69 countries and cycling data for 15 countries. Global air mobility in 2023 reached ≈94% of the pre-COVID-19 pandemic levels and was sourced from[64]. Biomass movement while walking or cycling was estimated based on global cellphone data on the number of daily steps[67], data on walking duration for various African countries[52], direct estimates for South American countries[49], and several national studies (see file S1). The assembled dataset covers ≈95% of the global population for air travel, ≈55% for road transport, ≈85% for rail-based mobility, and ≈75% for walking. Our data-driven estimates of human mass movement amount to ≈3,200 Gt•km/yr.

We additionally modeled mobility data for the missing population via the average of two extrapolation approaches, adding another ≈1,200 Gt•km/yr to arrive at ≈4,400 Gt•km/yr with 1.3-fold uncertainty. For this additional modeling, we grouped all countries into standard economic income groups, as developed by the World Bank[10]: high income (HI), upper middle income (UMI), lower middle income (LMI), and low income (LI). In the first approach, we calculated the average traveled distance per capita for each mobility mode and each economic income group from the data. These average per capita values were then used to extrapolate the pkm/yr for all countries and populations in the same economic group for which no data was available. In the second approach, we extrapolated mobility estimates for all missing populations using a regression model based on GDP per capita as a predictor for each mode of transportation. The reported uncertainty represents the extreme values between the second modeling and two standard errors of the mean (SEM) of the per capita passenger-kilometer (pkm). We added a ten percent uncertainty to the mobility of countries with data.

Our primary estimates calculate human biomass movement by multiplying the total pkm per income group by the average body mass of all people in that group[9]. However, the share of adults in transportation is likely larger than their relative share in the population, and globally, adult body mass is ≈20% larger than that of the general population (including children). We account for this possible bias by using the body mass of adults in our upper estimates. This also affects uncertainties in our human biomass movement. They are calculated for each transportation mode as half the differences between the upper and lower estimates.

Supplementary Table 1. Global human mobility estimates and uncertainties by mode of transport.

Transportation mode	Daily distance per capita [km]	Total Biomass movement [Gt•km/yr]
Road	17 ± 4	3,000 ± 1,000
Rail	1 ± 0.1	210 ± 50
Walking and cycling	5 ± 2	800 ± 500
Air	3 ± 0.4	500 ± 100
All modes	27 ± 5	4,000 ± 1,000

The total biomass movement uncertainty is calculated as the root mean square of the individual uncertainties (see file S1).

Supplementary Table 1 and Supplementary Figure 1 summarize our global human mobility estimates. We find that nowadays, human biomass movement is dominated by road mobility (≈65%), followed by walking and cycling (≈20%) and air travel (≈10%). Rail-based mobility makes up only ≈5% of global human biomass movement (see Fig. 3 of main text). Land mobility per capita varies significantly between income groups (Supplementary Figure 1), with people in high-income countries traveling most (≈42±6 km/cap/day), people in upper-middle income countries (≈25±6 km/cap/day) and lower-middle income countries (≈18±6 km/cap/day) significantly less and people in low-income countries being the least mobile (≈12±11 km/cap/day), partly due to a lack of infrastructure[40]. However, the majority of people live in countries with upper-middle and lower-middle income economies, and they together contribute most of the human biomass movement (≈63%). Supplementary Figure 2 compares our final biomass movement estimates, including extrapolations, to the reported data. Both data types have similar per capita biomass movement per income group and transportation mode, with varying data coverage, as shown in Supplementary Table 2. The data coverage is larger for motorized mobility and for high-income countries.

Active mobility includes both walking and cycling. Walking estimates were calculated using the general method outlined above. We calculated cycling estimates based on the relative proportion between cycling pkm to walking pkm in 15 countries with both cycling and walking trips data. The combined cycling pkm was found to be about 30% of the combined active pkm in all 15 countries, which was used as our best estimate for extrapolation. Alternative extrapolation assumptions extended the uncertainty range of these estimates (see file S1).

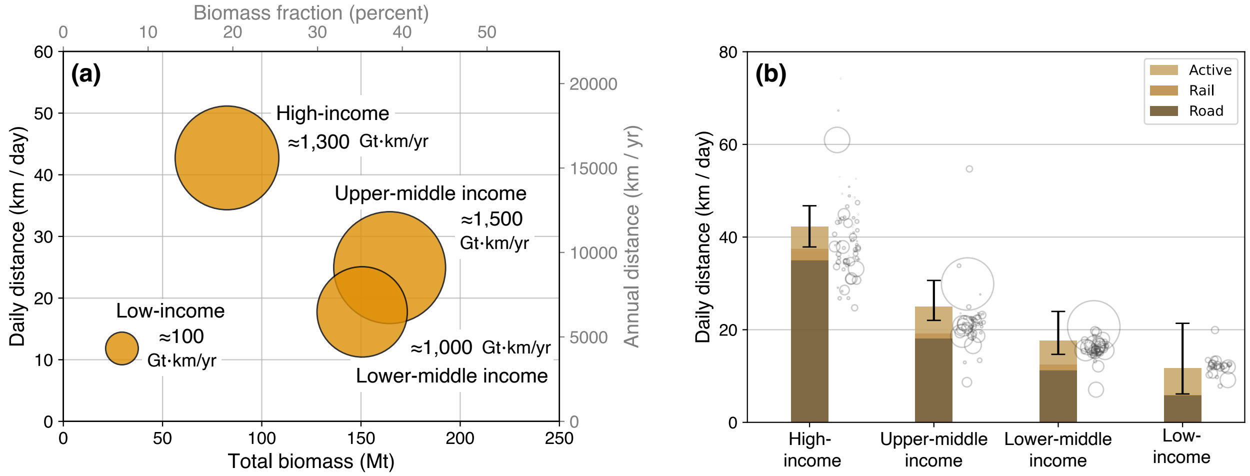

Supplementary Figure 1. Comparison of human terrestrial biomass movement across economic income groups. (a) Breakdown of total biomass movement. High-income, upper-middle-income, and lower-middle-income groups contribute similar amounts to the global human biomass movement, with low-income economies responsible for only ≈3%. High-income economies move more per capita, but their population is smaller than that of upper-middle and lower-middle-income economies. Biomass movement due to airborne and waterborne transport is excluded from this figure due to a lack of data for allocation into income groups. (b) Daily distance traveled by mode of transportation and income group. Circles represent n=217 country or territory-specific data whose population-weighted average forms the total bar heights. The size of each circle is proportional to the population of the corresponding country or territory.

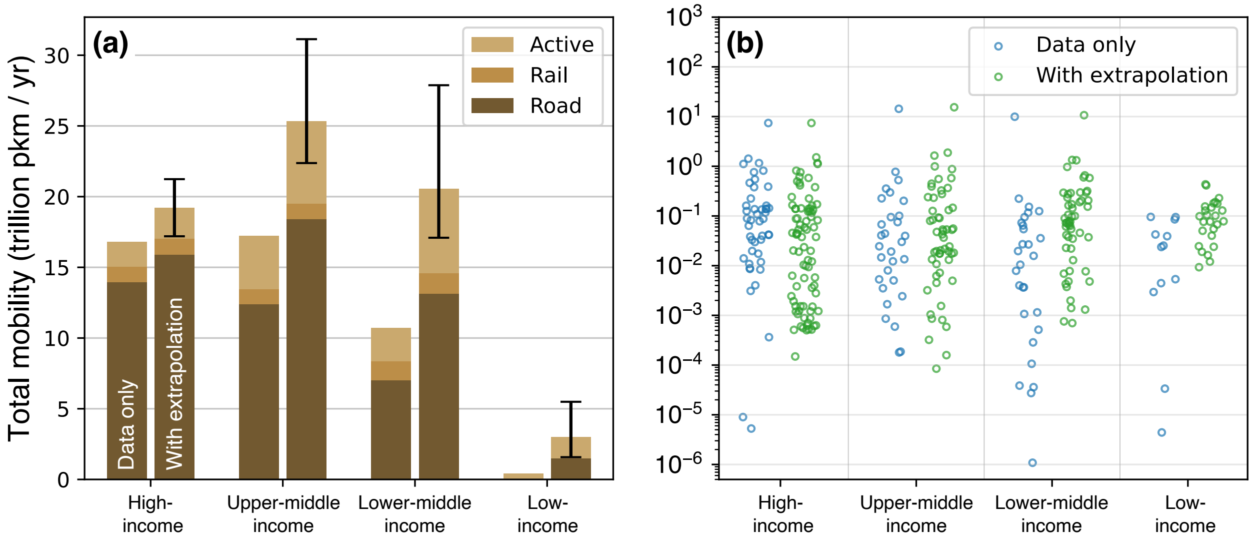

Supplementary Figure 2. Comparison of human terrestrial mobility across economic income groups. (a) The left bars represent passenger-kilometers calculated directly from our data. The right bars represent passenger-kilometers based on data and extrapolations, covering all human population. (b) Underlying country or territory-specific total mobility. Supplementary Table 2 presents the sample sizes and population data coverage.

Our data on walking might be biased towards transport or long trips and miss shorter trips and everyday walking within work- and living environments. Our estimates for walking agree with the lower range of typical data from pedometers. Our global average is ≈4 km/day walking (range 2.9-4.3 km/day), equivalent to ≈5,000 steps per day. In comparison, adults which move less than 5,000 steps per day may be considered 'sedentary'[65]. Similarly, normative pedometer data suggest that adults under 65 take ≈5,000-10,000 steps per day in various high-income countries[65], with some data suggesting that children and adolescents may walk longer distances than adults[66].

Supplementary Table 2. Breakdown of data coverage on human mobility.
We used extrapolation and regression models to account for populations not covered by our data. Income groups naming scheme: HI: high-income economies; UMI: upper-middle-income economies; LMI: lower-middle-income economies; LI: low-income economies.

Transport mode	Income group	Countries covered	Population covered [%]
Road	HI	35	85%
	UMI	12	62%
	LMI	3	47%
	LI	0	0%
Rail	HI	40	95%
	UMI	28	95%
	LMI	26	84%
	LI	6	32%
Active	HI	32	94%
	UMI	16	94%
	LMI	12	61%
	LI	9	45%
Air	Global	105	96%

Estimate of human biomass movement in 1850

We find that human biomass movement in 1850 was ≈40 times smaller than it is today. The two most significant factors for the reduced biomass movement relative to today are a ≈6 times smaller global population, and the lack of motorized cars and airplanes. The increase in the average human body mass due to aging and fattening of the global population[9] has increased human biomass movement by an additional ≈30% since 1850. We used different approaches to approximate biomass movement across all modes of transportation, and found that walking constituted most of human biomass movement in 1850. We describe here the general process, but refer to file S2 for further details.

In 1850, air travel was negligible in terms of biomass movement, as it consisted mainly of balloons, and airplanes were not invented yet. Road mobility consisted only of horse carriages, as the modern car was not invented yet. From an average of ≈3 million yearly trips in the UK[68] and assuming trip lengths between 80 km and 160 km, we calculated total preson-km traveled in the UK per year. This figure was then upscaled to reach a global estimate of ≈5 billion pkm/yr using historic population data[69], and taking into account the difference in urbanization levels in the UK in 1850 (≈40%) compared to the global average (≈12%).

Rail travel was relatively developed in several countries in 1850 (USA, UK, Ireland, Germany, France, Austria, Belgium, Russia and the Netherlands), with a combined population of ≈180 million people and a combined network length of ≈40,000 km. This is ≈2% of the current global population, and ≈10% of the current railway network length in these countries, or ≈3% of the global railway network length. We estimate the current average rail mobility as ≈500 km/cap/yr, and ≈4 trillion pkm/yr. If we assume that the intensity of rail use in 1850 was as large as it is today per capita or per railway network length, we find it to be ≈2-3% of today's value, or ≈100 billion pkm/yr. This is an upper bound for rail transport in 1850. However, we may assume that a country that had 10% of the current railway network length also had a 10% per-capita rail mobility relative to today. With this assumption, that the per-capita rail mobility is directly proportional to the available railway network length in each country, we find that the ≈180 million people which had access to railways in 1850 were traveling an average of ≈200 km/cap/yr, or a total of ≈30 billion pkm/yr. Since the total global network length was ≈10% of the current global network, making the above assumption on the global scale would mean that ≈180 million people were traveling on average ≈50 km/cap/yr, with a total of ≈10 billion pkm/y. In reality this too might be an overestimate. To test this, we estimate the intensity of rail use in 1850 using UK figures. The UK had an extensive rail system, with about a quarter of the global railways infrastructure in length, and ≈75 million rail passenger-trips[68]. Its population was ≈40% of the country's current population, but the number of passenger-trips per year was ≈4% of the current figure, of ≈1,700 million passenger-trips per year in 2014-2019[70]. Assuming similar trip-distance as today (despite trips taking much longer times in 1850), means that the rail mobility per capita in 1850 UK was ≈10% of the current figure, or ≈100 km/cap/yr. Applying this figure

to the UK in 1850 reduces the total rail mobility estimate to ≈20 billion pkm/yr. The range of our estimates is thus 10-100 billion pkm/yr. However, since trains are faster and more affordable today, these figures might be an overestimate of the true value.

For horse riding we develop an upper bound estimate. The peak in the global horse population was at around 1920, with ≈100 million horses worldwide, including draft horses[71]. If we assume that at 1850 there were as many as 50 million transportation horses, and they each transported a person 20 km a day for 100 days a year, it gives ≈100 billion pkm/yr. Varying these estimates results in a 5-fold uncertainty.

We find that walking overwhelmingly dominates 1850 human mobility. To estimate 1850's active human mobility, we followed different approaches. In the first approach, we assumed the current active mobility per capita holds for 1850 as-well. We applied it for each current income group of countries and multiplied it by the historic populations. This resulted in an estimate of ≈2.5 trillion pkm/yr. In a second approach, we used 1) for an upper estimate a reported number of ≈3,000 km/cap/yr of active movement in rural areas is Austria around that time[72] and 2) for a lower estimate the current estimate for walking alone ≈1,400 km/cap/yr and multiplied it by the historic rural population[73,74]. The urban active mobility was derived from today's data, based on all active movement (upper estimate), or only walking (lower estimate). The two estimates were calculated at ≈2-4 trillion pkm/yr. Our third approach was based on the assumption that travel time is constant over time, and thus in 1850 about the same as today's reported ≈1 hour/day[75]. By multiplying this duration with an average walking speed of ≈4 ± 1 meters per hour, which was calculated from Refs.[42,76–78], we found an average distance of ≈1,500 km/yr per capita. Extrapolating this on the global human population in 1850 resulted in a global active mobility of ≈2 trillion pkm/yr. The average from all three approaches led to a result of ≈2.4 trillion pkm/yr traveled by active mobility (walking and running) in 1850, with a range of 1.5 - 4 trillion pkm/yr. In total, we found that in 1850 humans traveled ≈3 trillion pkm/yr. This corresponds to a global biomass movement of ≈100 Gt•km/yr with an uncertainty range of 60-200 Gt•km/y. Over 90% of this biomass movement was being traveled by foot.

Mass movement of vehicles

In this section we extend our focus to include not only human biomass movement as done throughout the estimates above, but also any vehicle mass-movement involved in moving humans. We compiled a number of scientific studies and other sources to estimate the number and the weight of passenger vehicles globally. These included cars, motorcycles and mopeds, railway coaches, trams, subway cars, buses, bicycles and airplanes (for details and sources we refer to file S3). This resulted in an estimate of ≈2 Gt for the combined mass of all vehicles in use, with over 90% of that mass found in light-duty cars. There are ≈1.3 billion cars in the world[79], with an average mass of ≈1.5 tons[80]. We found that light duty cars comprise ≈70% of all passenger vehicles mass-movement, with aircrafts contributing ≈15% (see file S3).

For calculating the annual vehicle movement (in units of Gt•km/yr), we multiplied vehicle masses with their annual mileage when such data was available, and combined it with additional estimates. We also calculated annual vehicle movement by multiplying vehicle masses with yearly pkm, dividing by average occupancy for each transport mode. For rail-based transport, an average rail vehicle and its occupancy were calculated from data on subway cars, railway coaches and trams, weighted by the number of vehicles of each type. These average rail vehicle mass and occupancy were then multiplied by pkm of rail mobility, resulting in ≈1,000 Gt•km/yr for the movement of rail vehicles. For bicycles, we used our estimate for cycling pkm, resulting in ≈80 Gt•km/yr.

We splitted the pkm of road mobility into two-wheeled, car and bus traffic. We followed two different approaches to estimate the total vehicle movement of road transport. First, we multiplied the total mass of cars by the average vehicle-km[81], and did the same for motorcycles/mopeds, assuming a vehicle-kilometer (vkm) of 10,000 km/year, as our lowest estimate for cars. For buses, we assumed 20% of road traffic being public transport, based on data from the World Bank[82]. This approach resulted in a total of ≈30,000 Gt•km/yr of vehicle movement for light duty road transport. In another approach we converted the human road mobility (in pkm) to vehicle mass movement using average masses for the different vehicles, average occupancies, and assuming 20 percent of pkm in public transport. This approach resulted in ≈40,000 Gt•km/yr for road transport. The average of both approaches was taken as our best estimate.

Airplane mass-movement was estimated similarly at ≈6,000 ± 2,000 Gt•km/yr. The estimate is based on estimating the amount of airplane-km/yr, and multiplying it by the average mass of an airplane. In one approach we divided the ≈8.5 trillion global revenue pkm (RPK) estimated by ICAO in 2019[62] by the average occupancy of ≈135 passengers per airplane according to ICAO[62] and the US Federal Aviation Administration (FAA) aircraft capacity and utilization factors[83], resulting in about 64 billion airplane-km/yr. A similar estimate of about 65 billion airplane-km/yr was made based on seat capacity of 160 seats per aircraft and ≈10 trillion seat-km in 2019[62]. A

third, less reliable estimate is based on average speed of ≈600 km/h and ≈10 block hours per day in US airplanes. It results in ≈2.2 million km/yr per aircraft, and ≈40 billion airplane-km/yr. Using an estimate for the amount of active airplanes by type in 2020[84] and the maximum take-off weight (MTOW) and maximum landing weight (MLW) for each airplane, we estimate their average MTOW to be ≈110 tons, and the average MLW ≈90 tons. Over half of the active airplanes are from the Airbus A320 family and Boeing 737 family, which are also at the center of the mass range distribution for commercial aircrafts, with MTOW of ≈70-80 tons. Assuming 135 passengers per airplane, the biomass of passengers is ≈8 tons per airplane. We thus estimate the average airplane mass as being ≈90 tons with a range of 70 - 120 tons, resulting in the above total estimate. This estimate agrees with the value obtained when multiplying the estimated biomass movement in commercial flights by the estimated aircraft-to-passenger mass ratio.

The total movement of all passenger vehicle mass was estimated at ≈40,000 ± 10,000 Gt•km/yr, dominated by light-duty cars. Freight transport was estimated to move ≈150,000 - 200,000 Gt•km/yr[18,85,86], dominated by maritime transport. International maritime trade alone is transporting over 10 Gt/yr about 60,000 billion ton-miles[18], which is ≈110,000 Gt•km/yr. Fossil fuels and raw materials such as iron ore and metals account for roughly half of the maritime transport.

Supplementary Table 3: Global passenger vehicles mass movement estimates by mode of transport. Light-duty cars account for over 90% of the mass of passenger vehicles and ≈70% of their mass movement. Airplanes have the second largest mass movement of passenger vehicles, accounting for ≈20% of the total.

Transportation mode	Total mass [Mt]	Total vehicle mass movement [thousand Gt•km/yr]
Light-duty vehicles	2,000	30
Buses	50	3
2-3 wheeled vehicles	90	1
Bicycles	40	0.1
Rail vehicles	7	1
Airplanes	3	6
Total		40±10

Wild terrestrial mammals excluding bats

We follow Greenspoon et al.[7] when defining wild mammals, excluding the pets domestic cat (*Felis catus*) and domestic dog (*Canis familiaris*), the synanthropic mammals: black and brown rats - *Rattus rattus* and *Rattus norvegicus*, respectively, and house mouse - *Mus musculus*, and livestock such as cow and sheep. Also following Greenspoon et al.[7], we excluded mammal species for which data such as range size are unavailable due to scarcity and lack of research, under the assumption that their biomass (and biomass movement) contribution is negligible compared to the rest of the mammals. This left us with ≈4,800 mammal species out of the known ≈6,400. About 900 of these species are bats, which are treated separately. We estimated the biomass movement of land mammals using measured daily distances for ≈200 species and an established model for daily movement distance (R) based on adult body mass (M)[87,88]. The general model is $R=a \cdot M^b$, where R is in units of km, M is in kg, and b=0.3 with range b=0.23 - 0.35. The data for body mass and global biomass of all the species of land mammals were obtained from[7]. The values for b were taken as best fit and range of the modeled daily movement in[87] to obtain the various estimates. We use order-specific parameters for Artiodactyla, Carnivora, Rodentia, and Primates, for which we have enough data. For our best estimate, we took the measured daily distances from Refs.[11,87,89–92] for species with such measured distances.

For the lower and upper estimates, we took the minimal and maximal modeled daily movement distances for each species, respectively. The measurements in[11] represent terrestrial mammals known to travel long distances, and we took the average of the reported total movement for each species. Supplementary Figure 3 depicts the biomass movement of wild land mammals by species. We found the total biomass movement for all land mammals to be ≈30 Gt•km/yr, with a full range of ≈10-70 Gt•km/yr. This biomass movement corresponds to a ≈3.7 km/day biomass-averaged movement for all wild terrestrial mammals (range ≈2-5 km/day). The combined biomass movement of the species with measured traveling distances was ≈20 Gt•km/yr, as they include species with long movements and large global biomass.

The ten species that contribute most to the biomass movement of land mammals correspond to ≈60% of the total biomass movement of land mammals and ≈35% of the total biomass (These ten species are: African savanna elephant, white-tailed deer, African forest elephant, wild boar, caribou, blue wildebeest, red deer, Eastern gray kangaroo, red kangaroo, and moose). We note that pigs and wild boars belong to the same species (*Sus scrofa*), and the biomass of feral pigs is significant[7]. We took as our best estimate for the biomass of wild Sus scrofa, the biomass found in their original range, being ≈0.6 Mt, thus excluding feral pigs in Australia and America. Our upper estimate for *Sus scrofa* includes the global population of feral pigs as estimated in[7], with a total biomass of ≈2 Mt. This inclusion of feral pigs would make *Sus scrofa* the third largest land mammal contributor of biomass movement, with a value of ≈3 Gt•km/yr, accounting for ≈10% of the total biomass movement of land mammals.

We also calculate an upper bound for the biomass movement of wild mammals being ≈150 Gt•km/yr (see 'Sensitivity and uncertainty analysis' section below for further details).

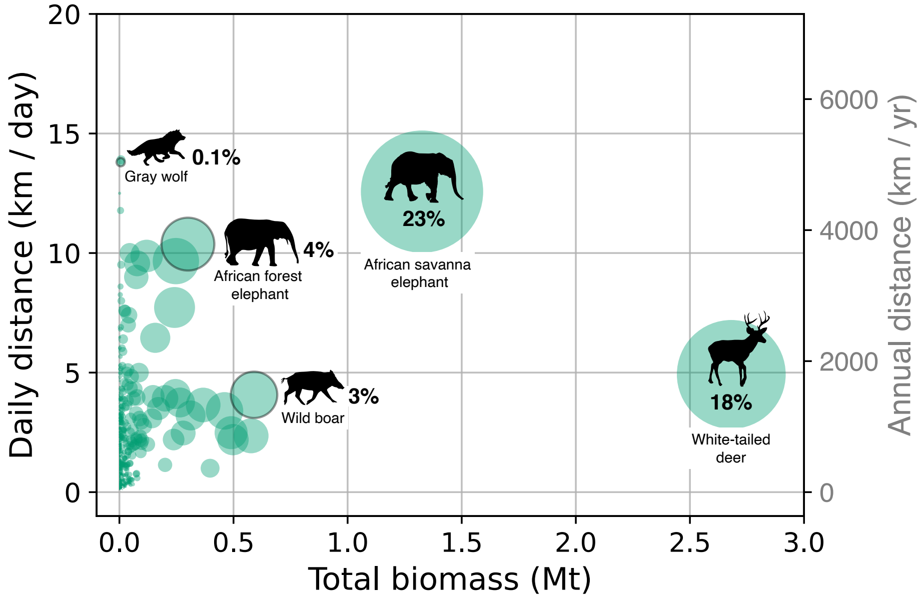

Supplementary Figure 3. Estimates for the biomass movement of wild land mammals by species. The top three contributors are the African savanna elephant, white-tailed deer, and African forest elephant, which contribute ≈23%, ≈18%, and ≈4% to the total biomass movement of all wild land mammals excluding bats, respectively. Wild boar (*Sus scrofa*) have a biomass movement of ≈3% of the total, excluding feral pigs. If we were to include feral pigs in the category of wild mammals, the biomass movement contribution of *Sus scrofa* would be ≈10% of the total. The gray wolf is a terrestrial mammal with especially long-distance recorded annual movements, also shown in Fig. 2. N=3,878 species are presented, with n=186 species having measurements of daily distances, and n=392 species having global population reports[7]. The spotted hyena are not shown, as their daily travel distance is larger than 20 km/day but their total biomass is small. Icons are from www.phylopic.org. African forest elephant icon is by Richard Ruggiero, vectorized by Julián Bayona and published under license Attribution-ShareAlike 3.0 Unported. Other icons were published under license CC0 1.0 Universal Public Domain Dedication.

Mammalian biomass movement during the late Pleistocene

We provide a very rough estimate of the historic biomass movement of the late Pleistocene megafauna. According to Barnosky[15], the biomass of wild land mammals that weighed >44 kg (megafauna) has seen a gradual decline starting ≈50,000 years before present, and crashed ≈12,000 years ago as part of the Quaternary Megafauna Extinction. This has happened following human expansion and as a rapid growth in human biomass has started to occur. During the time period 20-100 thousand years ago, the biomass of wild megafauna was estimated by Barnosky[15] to be ≈0.2 Gt, which is ten times larger than the current biomass of wild land mammals. Most of the extinct mammals were megafauna[227,228].

We repeated the calculations of Barnosky and extended them to all historic land mammals. The total biomass estimate is about 0.05 Gt larger than that of megafauna alone, but it remains ≈0.2

Gt at the single significant digit level, with ≈90% of it in herbivores. We estimated the daily movement of each species using the general allometric relations of[87] and their uncertainty range. We find that the total biomass movement of wild mammals was ≈400±200 Gt•km/yr, with almost all of this biomass movement being made by herbivores.

We note that Barnosky[15] did not provide an uncertainty range to the biomass estimate. The main sources of uncertainty originate from the population estimates, which in turn are based on rough estimates for the historic range sizes of mammals and rough allometric relations for their density[229]. The range sizes differ significantly with latitude, and their distribution is heavily left-skewed[230]. While the average continental area for herbivores of the Palearctic was measured at ≈8%[230], as was assumed by Barnosky[15], the median in[230] for the various groups is a few percent, and the range of some herbivores extends to ≈30% of the Palearctic. We roughly estimate that this can potentially introduce a multiplicative uncertainty of ≈3. The multiplicative uncertainty in the population density is ≈10-100 fold for each herbivore body mass[229]. For large carnivores this uncertainty is ≈5-fold. To get a rough understanding of how significant this bias could be, we roughly estimate an upper value for the log(density) relation with log(body mass) by fitting a linear line to the herbivores data points in Fig. 3 roughly half-way upwards between the fitted trend line and our visual estimate of the 95% prediction interval. The equation for this fitted line is log(density in number per square kilometer) = -0.67 log(body mass in kg) + 2.22. Using this relation results in an upper biomass estimate of ≈1 Gt, and a biomass movement of up to ≈2,000 Gt•km/yr. To get an upper bound, we roughly fit a line to the prediction interval having an equation: log(density in number per square kilometer) = -0.70 log(body mass in kg) + 2.66. This resulted in an upper bound biomass estimate of ≈2.5 Gt, and an upper bound for the biomass movement of ≈5,000 Gt•km/yr.

It is possible that the average population densities were lower than the ones measured[231]. We tentatively estimate the lower range of the biomass of wild mammals 100,000 years ago as ≈50 Mt, roughly twice the current mammalian biomass, acknowledging that the massive megafaunal extinctions and reductions since that time are likely to have significantly reduced the total biomass of wild mammals. We therefore very roughly estimate a tentative uncertainty range of ≈50-2,000 Mt, and a tentative biomass movement range of ≈100-2,000 Gt•km/yr, with an upper bound of ≈5,000 Gt•km/yr.

The human population during the Pleistocene is estimated at under ten million people[93], resulting in human biomass movement of less than 1 Gt•km/yr, under the assumption of about 5 km/day and less than 50 kg/person[15].

Wild birds

We estimate the biomass of wild birds based on population estimates of ≈3,900 bird species from BirdLife international[89], body-mass, species range, taxonomy, extinction risk, and traits data for all bird species[90]. We estimate the average yearly distance traveled by each bird species using tracked movement data for ≈5,800 individual birds of ≈135 species from Movebank[91], and an additional 15 species from the literature.

Biomass estimate:

The biomass estimate of birds was estimated by Bar-On et.al. to be ≈12 Mt[13], based on two independent estimates of 6 Mt and 24 Mt. However, these estimates were based on very limited data and contain a large uncertainty that was not evaluated. We used more detailed species-specific data from BirdLife[94] that included abundance range estimates for ≈3,900 wild bird species out of the ≈11,000 extant species, and combined it with body-mass data[95] to estimate the total biomass of these ≈3,900 species. Their total biomass was found to be ≈2-3 Mt, corresponding to the sum of the reported minimal population estimates and the sum of the reported maximal population estimates. Our best estimate for the total biomass of these ≈3,900 bird species is taken as the geometric mean of the two, at ≈2.2 Mt.

The ≈3,900 species with global population estimates contain some of the most abundant and massive bird species on Earth, but also many rare species. About half of the 3,900 species are evaluated as least concern (LC) by the IUCN red-list. This half contributes ≈85% of the total biomass estimated for the 3,900 species. About 90% of the bird species without population estimates are also categorized as LC.

For each species we estimated its total biomass as a lower and upper estimate, and their geometric mean was our best estimate. For the 3,900 species with BirdLife population estimates, the biomass estimate was the direct multiplication between the corresponding population estimates and the species body mass.

We trained a Random Forest algorithm on a random sample of 90% of the species with known biomass, range, taxonomy, extinction risk, and traits, and repeated it ten times for cross validation. The total biomass was found to be in good agreement. Training the data on all the species with biomass estimates and extrapolating to the remaining species, we found the total biomass of all birds to be ≈3 Mt. Repeating this procedure for the upper and lower estimates of the 3,900 species, we found a total biomass range of 2.5 - 5 Mt. This is smaller than the crude estimate of[13].

As a sanity check, we also use a naive estimation approach. We calculated the average biomass of the 3,900 species divided into groups by their IUCN extinction risk category, and by their

taxonomic order. These averages were assigned as the biomass per species to all the remaining species, for which we didn't have population estimates, according to the groups they belong to. 65 bird species were in groups where no biomass estimates were available, and we assigned them an average biomass based on their taxonomic order alone.

We also made alternative estimates by repeating this procedure while grouping the birds also by the migration category of each species[95], or by excluding the extinction risk category or order in the grouping stage.

We found a total biomass estimate of ≈7 Mt with an estimation range of 4-12 Mt across the alternative estimates. This range agrees with the range found above, but is higher due to the effect of species with exceptionally large biomass. Removing from this procedure 134 species with exceptionally high biomass (being over 2 standard deviations away from the mean of their extinction risk and order group), representing 1 Mt in total, we find the total biomass to be in the range 4 - 7 Mt when grouping by extinction risk and order, and 3-5 Mt when grouping by taxonomic order alone. This agrees well with the above estimate based on a Random Forest regression, the ≈4 Mt estimate of[97], and the lower estimate of [13].

Biomass movement estimate:

We are not aware of any study that estimates the average daily distance traveled by all birds, or any general relations from which such estimates could be deduced. Therefore we estimated such distances based on publicly available raw tracking data. We estimated the total distance traveled based on data from Movebank[91].

Data filtration. We downloaded all freely accessible telemetry data from Movebank, and filtered all studies of wild birds (class Aves) based on the species list of BirdTree[98]. We removed measurements that used a solar geolocator, due to high inaccuracy in their location measurements which resulted in biased traveling distance estimates. Next we calculated the time and geodesic distance passed by each bird between consecutive position measurements, and the average speed for each such incremental movement. Differences between consecutive latitude and longitude were also calculated. We filtered out outlier incremental movements that were suspected to be unrealistic: We excluded incremental movements that exceeded a speed of 100 km/h, and geodesic distances or coordinate position differences that were at least 5 standard deviations larger than the average incremental distance moved by each bird. This removed less than one percent of the positions in the dataset. We also filtered out studies according to the following criteria: Only measurements of sensors with sampling intervals of less than 24 h were accepted. For measurements of species belonging to the migratory and partially-migratory groups[95], only measurements of duration longer than a year were retained, to avoid measurements focused only on migration times. This left us with telemetry data for ≈6,000 individual birds of 135 species.

Traveling distance and biomass movement estimates. The total biomass movement of each species was calculated as the product of its measured or estimated yearly traveling distance and its biomass. This calculation was repeated for alternative biomass estimates and alternative traveling distance estimates to acquire an uncertainty range. The total biomass movement for all wild birds was found to be ≈30 Gt•km/yr with a 2-fold uncertainty estimate. About a third of the total biomass movement is due to birds whose primary lifestyle is aquatic, including seabirds like petrels, albatrosses, penguins, and waterfowls. Supplementary Figure 4 depicts the biomass movement of all birds by taxonomic order.

We started by calculating a probability distribution of the average daily distances traveled by each bird species, by bootstrapping the individual birds of each species in the filtered dataset. We combine these distributions to form probability distributions for the daily distances of each species group having a shared migration category, primary lifestyle, and taxonomic order[95]. These distributions cover ≈70% of the bird species, and ≈70% of the total bird biomass. We repeat this procedure using broader groupings, with fewer variables, to cover the rest of the species (i.e. using only taxonomic order and migrations category, followed by using only the order and only the migration category). From each distribution, the 2.5 and 97.5 percentiles, as well as the mean, is extracted. We used these means to estimate the average daily distance of the other species in each group (for which we have no telemetry data). The percentiles are similarly used to extract an upper and lower estimate for each group. This resulted in an overall biomass-weighted movement estimate of ≈30 km/day.

We also surveyed the published literature for reported estimates that could complement our daily distance estimates based on raw tracking data. We focused our survey on birds from taxonomic orders with relatively large biomass, and on birds from groups that are poorly covered in our dataset. For example, we estimate that galliformes (gamebirds) constitute about ten percent of the total biomass of wild birds, but our movement dataset only includes data for a single galliform species. We found relevant estimates for 15 additional bird species, including four galliform species. Including these species does not change much the overall estimate.

We note that we did not include the data reported by Cooper and Marra[99]. They have reviewed the literature and estimated the daily traveling distances of 47 bird species. Most of their data is derived from foray studies and studies of alternate space-use, and much of it is based on assumed ratios between daily distance moved to territory or home-range size. These estimates were not found compatible with our metric, hence we did not include them in our estimate. Including the reported distances in[99] has a minor effect on the overall estimates, and its main effect is to reduce the typical daily distance calculated for songbirds.

We note that despite our filtering, our small bird species sample from which we infer the daily distances may be biased in various ways[6]. The telemetry measurements over-represent larger birds, and may include additional biases such as a resolution bias that causes us to under-estimate the true distance moved[6,100,101]. However, this resolution bias is reduced when a bird moves in a relatively straight path (having a low tortuosity), and noise in the measured positions may inflate the extracted distance moved[101].

To get an upper estimate for the daily distance birds move, we concentrate on migration speed data of 115 bird species from 35 families of 14 orders[102]. The migration speed of most species was found to be in the range 20–100 km/day, with a median and average of ≈50 km/day. This corresponds to ≈60 Gt•km/yr assuming our best biomass estimate, which is within our 2-fold uncertainty. Applying the average migration speed to an upper biomass estimate of 5 Mt for all birds, results in an upper bound of ≈120 Gt•km/yr.

Supplementary Table 4. Wild birds data coverage (rounded).

Group	Number of species	Taxonomic coverage (% of species)	Total biomass (Mt)	Biomass coverage	Biomass mobility coverage
Species with measured distances	150	2%	0.6	20%	20%
Orders with measured distances	35 orders	90%	3	80%	70%
Species with evaluated population size	3900	35%	2	70%	80%
All wild birds (class: Aves)	11000	100%	3	100%	100%

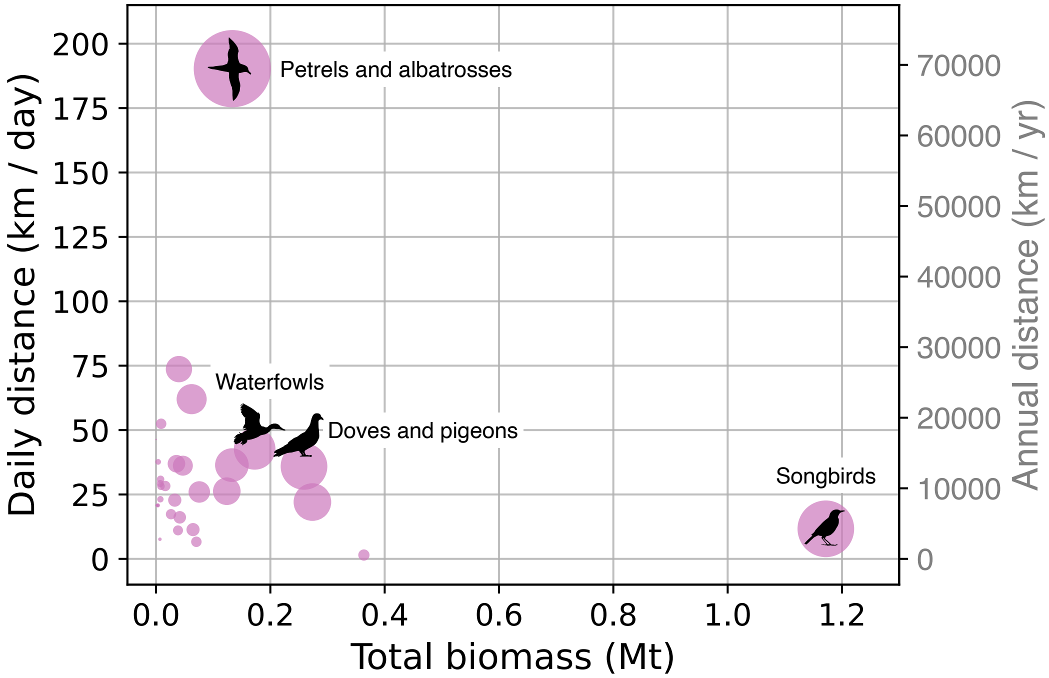

Supplementary Figure 4. Total biomass movement of wild birds by taxonomic order. Distances are biomass-weighted averages over all species in each taxonomic order over a year. The area of each circle is proportional to the total annual biomass movement of the corresponding order. Varying the extrapolation assumptions may significantly change the biomass movement of certain orders, but the combined biomass movement of all birds remains similar. The total biomass movement of all wild birds is ≈30 Gt•km/yr. N=35 orders are presented with n=149 species having measurements of daily distances, and n=3,874 species previously having global population estimates (see Supplementary Table 4). Icons are from www.phylopic.org under license CC0 1.0 Universal Public Domain Dedication.

Terrestrial arthropods

The total biomass of terrestrial arthropods is ≈ 300±200 Mt dry biomass[23], or ≈1 Gt wet biomass. About 70 percent of this biomass is found in the soil[23]. The soil medium is probably the one in which movement is the hardest. However, many of the arthropods considered here as soil arthropods also move outside the soil, such as foraging ants and termites, and in tunnels and pores. Terrestrial arthropods are extremely diverse, yet data regarding their daily movement is very limited, and much of their movement takes place in fine scales that may be hard to measure. Hence, the uncertainty of our estimates for terrestrial arthropods' biomass movement is high. Due to the lack of data and the variability in movement patterns, we evaluate minimal and maximal estimates for the different parameters (such as speed, biomass, and effective fraction of population that is moving) for each animal group, calculate a multiplicative uncertainty for each parameter, and take the geometric mean of the minimal and maximal values to be our best estimate for it. The multiplicative uncertainty of the total estimate is taken as the square root of the sum of squares of the multiplicative uncertainties, assuming log-normal distribution of the parameters. Since this group of animals represents the highest uncertainty in our data, we also estimate extreme upper bounds for it, and find that human biomass movement exceeds even these extreme values.

Termites

Most of the movement in termites is done by foraging workers and soldiers, and their movement is also affected by environmental conditions such as temperature and humidity, and by the geometry of the environment in which they move. The running speeds of termites in the family Rhinotermitidae were found to be about 50 - 120 cm/min[103,104], and 500±100 cm/min in laboratory conditions for the arboreal species *Nasutitermes corniger*[105]. Gautam et al.[106] found normal foraging speed to be 14 - 44 cm/min for the species *Reticulitermes santonensis* and for *R. hesperus*, respectively. We thus take their average speed to be at the range 30 - 200 cm/min, with the best estimate being its geometric mean of ≈80 cm/min. Also, Lee et al.[107] have found that about 20% of subterranean termites are constantly foraging, and we take the effective population size moving to be in the range 10-60%. We further assume that the fraction of time termites move (accounting for daily and seasonal variations) is within the range 20-80%. Their total wet biomass is ≈300 Mt with ≈2-fold uncertainty[23]. This results in a total biomass movement of ≈10 Gt•km/yr with a 5-fold uncertainty.

Ants

The running speed of ants is affected by their body size, temperature and other factors[108]. The running speed of ants is ≈1-20 cm/sec, with an average of ≈4 cm/sec. Most movement is made by foraging ants, which are not always active themselves. Their fraction was estimated to be in the range 5-22%[109] and we increased the upper range to 40% to include additional movements. We also estimate that ants are effectively active 30-80% of the time, being dominated by tropical

species. The global wet biomass of ants is ≈100 Mt[23,109] with a 2-fold uncertainty. This results in a total biomass movement of ≈10 Gt•km/yr with a 5-fold uncertainty.

Soil microarthropods

The biomass movement of soil microarthropods is very small. Their movement is typically restricted to within the pores in the soil[110]. Ojala and Huhta[111] found that microarthropods in Finnish forests, representing a biome with a significant share of the microarthropod population[23], have a potential dispersal rate of up to ≈20 cm/week, or 0.01 km/year. Lehmitz et al.[112] found dispersal of similar magnitudes. Using this upper dispersal rate for approximating the movement rate of all soil mites and springtails, having ≈150 Mt wet biomass[23], the total biomass movement of soil microarthropods is estimated at ≈1.5 Mt•km/yr. In order to account for the difference between dispersal rate and distance traveled, we approximate the movement of the microarthropods as a random walk with a straight step length having a typical value of ≈1 mm. Covering the maximal dispersal rate of 20 cm/week would require about 40,000 random steps[113], having a total path length of ≈40 m/week, or ≈2 km per year. Assuming the upper value for their biomass, ≈300 Mt, results in a total biomass movement of ≈0.6 Gt•km/yr, which serves as an upper estimate for the biomass movement of microarthropods.

The biomass movement of soil microarthropods is thus negligible with respect to other arthropods.

Other terrestrial arthropods

Other soil-related arthropods have wet biomass of ≈160 Mt, and another ≈160 Mt is found above-ground. Arboreal ants probably comprise tens of percent of above-ground arthropods[23] and were already considered separately, but the total biomass of above-ground arthropods may also be significantly under-evaluated. We take 160 Mt as the estimate for above ground arthropods that are other than ants and termites and with a 4-fold uncertainty[23].

Typically, most arthropod biomass is found in immature individuals, due to their high mortality rate and relatively long life-span. These juveniles, however, typically move much less than the adults. While adults typically need to reproduce and have developed locomotion capabilities, such as wings, juveniles typically keep to a small area and concentrate on feeding and growing. Therefore, as an upper estimate, we assume that around half of the ‘other’ arthropods biomass is made of juveniles that have much smaller total biomass movement than their adult populations, and hence can be neglected.

Many soil and plant-litter dwelling arthropods are relatively sedentary, being burrowed most of the time while moving relatively slightly. In addition, larger arthropods tend to move faster than smaller ones[114] and have lower cost of transportation relative to their body mass, allowing them

to actively travel greater distances. Large adult beetles were found to move up to tens of meters a day, and rarely cover a few hundreds of meters a day, e.g.,[115–117]. Therefore an average daily distance of 100 m can serve an upper estimate, with a 10 m estimate being more typical. Applying a 100 m estimate for all 'other soil arthropods', including juveniles, we find a total of ≈20 Gt•km/yr, while a more realistic estimate would be 0.3 Gt•km/yr with ≈10-fold uncertainty.

Other non-flying above-ground arthropods, such as Orthopterans,[118–122] are also presumed to move ≈tens of meters a day. We use an upper estimate of 200 m/day for half the biomass above-ground, and get ≈6 Gt•km/yr with a lower estimate of ≈1.5 Gt•km/yr**.** Flying insects may travel much greater distances, but probably have relatively low total biomass. As with other animals, larger flying insects have the capacity to actively travel longer distances. Some lepidopteran species take part in large migrations during which they can travel tens of kilometers a day or even more, with a total distance of thousands of kilometers a year[123–126]. However, as larvae they don't move much and only several percent of lepidoptera species are known to migrate[126,127]. Other flying insects were found to actively fly up to a few tens of km per day (and even longer in special cases and when assisted by wind, e.g.,[128]), but most arthropods typically move much less than their capacity, and a range of flying insects were found to travel or disperse typically up to a few hundreds of meters a day (e.g.,[129–137]) In addition, most migrating species are associated with temperate regions, whereas most above-ground arthropod biomass is found in tropical and subtropical regions, where they typically move less. Therefore, we assume that between 5-50% of the above-ground arthropods (by biomass) are flying, with an average daily distance of 0.2-3 km, resulting in ≈7 Gt•km/yr, with 5-fold uncertainty. We also assume that about 1 percent of this biomass covers as much as a thousand km a year (≈3 km/day), similar to the migrating bogong moths[123], which adds ≈2 Gt•km/yr.

Altogether, we find that the total active movement of terrestrial arthropods is ≈40 Gt•km/yr with a 4-fold uncertainty. It is possible that the biomass used for above-ground arthropods is under-evaluated[23], or that the fine-scale tortuosity of arthropod movement results in larger distances traveled. We account for such possible biases by using the upper bound biomass estimate for above-ground arthropods of[23], which is twice its evaluated upper range, and doubling the evaluated distances. This results in a biomass movement upper bound of ≈300 Gt•km/yr[23]. This is equivalent to all land arthropods traveling ≈1 km/day every day of the year. We also estimate an extreme upper bound for each group, detailed in Supplementary Table 12, by simultaneously taking the maximal plausible value of every uncertain quantity in the evaluation of the biomass movement of every land arthropod group: taking their maximal biomass estimates and the maximal estimate for annual distances or the maximal average speed and maximal activity times, resulting is combined estimate of ≈1,000 Gt•km/yr.

Upper bound estimates for other terrestrial animals

Earthworms are a dominant contributor to terrestrial annelids, with a total biomass of ≈200 MtC[13]. This is equivalent to ≈2,500 Mt wet biomass[138,139]. Bastardie et al. have found five species of earthworms to move 0.1-3 m/day in artificial soil cores[140]. Burrowing was found to be much slower than moving in existing pores and burrows, and most of the daily displacement was made while moving freely in pre-existing burrows. Earthworm displacement velocity was ≈40 cm/h for all examined species, and did not correlate with daily displacement. Using the upper value of 3 m/day over the entire annelids population yields an upper estimate of ≈3 Gt•km/yr.

Nematodes are numerically very abundant in the soil, but their typical length is only ≈0.3 -3 mm[110]. Their global wet biomass is ≈300 Mt[141]. Nematodes have various movement patterns, but in general were found to move ≈10 cm/day, with some found to move several tens of cm per day[142–148]. Assuming a total movement of as much as 50 cm/day yields an upper estimate of ≈0.05 Gt•km/yr for nematodes.

Amphibians and reptiles are ectotherms and most of them don't move as much as mammals of similar size due to their significantly lower metabolic requirements[149], need to thermoregulate, and more. Their body mass is typically small, with the vast majority of species having a body mass of less than 1 kg[150], resulting in a small biomass movement. The total biomass of reptiles was estimated in[13] to be ≈20 Mt, which is likely an overestimate. Even if we would assume they all move as much as a "characteristic" 1 kg terrestrial mammal, their total biomass movement would be ≈6 Gt•km/yr. We take 10 Gt•km/yr as an upper bound for reptile total biomass movement.

Sea turtles are the exception, and may even dominate the biomass movement of reptiles. They have a large body mass and swim vast distances. That said, their contribution is negligible with respect to other swimmers. The upper bound of the best estimate of global sea turtle population that we are aware of[151] is less than ten million adults, with over half of them being the relatively small species Olive ridley (*Lepidochelys olivacea*), weighing a few tens of kilograms. Assuming ten million adults with the upper range of their reported adult body mass per species results in a global biomass of <1.5 Mt. Assuming they all travel as much as 20,000 km/yr, their biomass movement would still be <30 Gt•km/yr. A more reasonable estimate based on[151] would be that their total global biomass is <0.7Mt (see 'file S6 - Sea_turtle_biomass.xlsx'), reducing their biomass movement to <14 Gt•km/yr. A more realistic traveling distance would reduce this estimate further.

There is currently no global biomass estimate of amphibians, and we have previously not found enough data to generate such an estimate[13]. We assume as an upper bound a total biomass of 20 Mt, representing a global average density of a few adult amphibians for every 10 square meters across a third of the ice-free land on Earth, being roughly the area of all the tropical, subtropical,

temperate, and flooded biomes, excluding croplands and pasturelands[152]. As an upper bound estimate for the average daily travel distance we assume 1 km/day despite the average amphibian weighing only several grams[13]. This results in an upper bound of 10 Gt•km/yr.

Likewise, terrestrial gastropods such as snails, a symbol of slow animals, are unlikely to have a biomass movement that is larger than that of terrestrial arthropods, which probably outweigh them also in biomass. Petersen and Luxton[139] note that while gastropods are among the most important groups in some ecosystems such as temperate deciduous forests, their biomass density is generally small compared to other soil invertebrates. We thus estimate their total biomass is less than 0.5 Gt, which is half the combined biomass of all terrestrial arthropods[23]. When active, they move at speeds of the order of up to a few mm per second[153,154], corresponding to typically up to several tens of meters per day. Therefore, we estimate that their total biomass movement is smaller than ≈10 Gt•km/yr.

We estimated the biomass of bats in a previous study[7] to be ≈1.4 Mt with a range of 0.7-3 Mt, although the data for bats populations is scarce. Bats move as part of their daily commutes, and some species undergo migrations that are typically shorter than those of birds[155]. In addition, many bats are known to hibernate during a period of the year, such as bats in temperate regions during the winter. We collected estimates for regular bat movements across 11 species of different characteristics[156–166], using measured duration of flight and flight speed, radio telemetry, or GPS. These estimates did not account for hibernation or migrations. The measured bats seem to travel on average less than ≈50 km/night. This figure holds for some reported migrations as well (e.g. [167]can be surpassed for certain colonies and during some migrations[155,167]. As an upper bound estimate, we thus assume that the biomass of bats is ≈3 Mt, and they all travel ≈50 km/night, resulting in an upper bound of ≈50 Gt•km/yr in total.

Supplementary Table 5: Summary of the biomass movement upper bound estimates of additional groups of land animals. See supplementary text for more details.

**Group**	**Wet biomass estimate [Mt]**	**Daily distance upper estimate [km/day]**	**Biomass movement upper bound [Gt•km/yr]**
Earthworms	2500	0.003	3
Nematodes	300	0.0005	0.05
Gastropods	<500	0.05	10
Amphibians	<20	1	10
Reptiles excluding sea turtles	≈20	1	10
Bats	0.7 - 3	50	50
**Total**			**<100**

Domesticated animals

The global biomass of mammalian livestock is ≈600 Mt, with roughly half being in non-dairy cattle[7]. Dairy cattle and buffaloes are the second and third largest contributors of biomass, respectively, with a combined share of ≈25% of the total[7]. Pets, including feral dogs and cats, contribute another ≈25 Mt[7], and poultry contribute ≈30 Mt[13]. While much of the chickens and swines in the world are being raised in intensive confined production systems (and hence have limited biomass movement), this is not the case for ruminants[168]. Land based systems, where the ruminants graze with or without additional farming, support about 1.5 billion cattle and buffalos across the globe and a similar amount of sheep and goats, mostly in developing countries[168]. Despite regions like North America growing most of their cattle in industrial feedlots, most non-dairy cattle in the world are grown in land-based systems[168]. Non-dairy cattle and buffalo represent ≈400 Mt and dominate the biomass-movement of domesticated animals. Sheeps and goats contribute ≈70 Mt[7]. Dairy cattle (≈90 Mt) are being grown in systems with various levels of confinement[168]. We assume that ≈400 Mt of cattle and buffalo are being grown in various land-based systems.

The daily traveling distance of non-dairy cattle as averaged over multiple studies seems to be ≈7 km/day, with a range of 2-15 km/day and standard deviation of ≈4 km/day[169–174]. Applying these values to all non-dairy cattle and buffalos places their global biomass movement at ≈1,000±600 Gt•km/yr. However, their movement might be shorter in many systems where the cattle are being

provided with a significant share of their diet in a concentrated small area. Assuming ≈2-6 km/day for sheeps and goats[175,176], they contribute ≈50-150 Gt•km/yr. Other domesticated animal contributions are neglected, due to their relatively small total biomass which limits their biomass movement.

The extent of live transport of animals in vehicles is uncertain, but we estimate that it is smaller than livestock actively moving. International live transport is dominated by cattle and pigs, each with ≈3-4 Mt/yr being transported[177]. Domestic live transport is also probably dominated by cattle and pigs, and usually include transport of calves or older cows to feedlots, and transport of adults to slaughterhouses. Trips by road typically span several hundreds of km or less[178–180]. As a very crude upper estimate, we assume that international live transport has an average distance of less than 7000 km, contributing up to 50 Gt•km/yr. We further assume that all ruminants and pigs are being domestically transported an average of between 100 to 1000 km/yr, mainly to slaughterhouses, contributing another 60-600 Gt•km/yr. We note that not all animals are being transported every year. Also, the transportation distance to confined feedlots is of similar magnitude as the distance that such cows would have walked if they were to continue grazing outside, therefore limiting the effect of this practice on the magnitude of the estimated biomass movement.

All in all, we estimate the total biomass movement of domesticated animals as ≈1,000 Gt•km/yr with a range of 400 - 2,000 Gt•km/yr, being dominated by cattle.

Marine animals

Marine zooplankton

According to[13], the biomass of mesozooplankton (size range: 200-2000 μm) is ≈0.7 GtC, of which ≈80% or ≈0.6GtC are arthropods. Macrozooplankton (size range: 2-10 mm) are ≈0.5 GtC, of which ≈0.15 GtC are pteropods and ≈0.04GtC are gelatinous. The total arthropod biomass is ≈0.9 GtC, and total zooplankton biomass ≈1.2 GtC. This corresponds to ≈7 Gt wet mass assuming 70% water content. However, more recent estimates[12] have placed the total biomass of mesozooplankton and macrozooplankton at ≈17 Gt wet biomass, of which ≈10 Gt are mesopelagic. The uncertainty of these estimates is ≈8-fold for mesozooplankton, and ≈11-fold for macrozooplankton. Smaller zooplankton (with body-mass in the range 0.1 pg - 10 μg) are neglected, since they rely on passive movement, or that their active mobility is very slow and hence small.

We estimate the average distance traveled by zooplankton in different ways.
First, we estimate their average swimming velocity from the general relation between body-length and cruising velocity[181,182], and relations between body-mass and body-length[183]. We apply this for each group in the biomass spectrum[12], and assume that at any given time ≈0.5 of

all zooplankton are active (with 2-fold uncertainty). We also limit the maximal average velocity of plankton at ten cm/s, being roughly the maximal velocity of krill. Many larger planktons are slow or even sessile, and thus don't obey the allometric relation used for swimming speed as a function of body length. This results in an estimated total biomass movement of ≈4,500 Gt•km/yr in the pristine ocean, with a minor reduction today, and with ≈3,000 Gt•km/yr due to mesozooplankton. This might be an overestimate, since the biomass spectrum in[12] assumes equal biomass for all body-mass range sizes, where it is likely to be more concentrated in smaller lengths and body-masses[184], for instance due to the dominance of copepods[13]. About half of the biomass movement of mesozooplankton is due to animals with body-mass larger than 100 mg, despite being at the upper end of their size spectrum. Assuming a few mm/s velocity for mesozooplankton, and a few cm/s velocity for macrozooplankton[181] results in a total biomass estimate of ≈1,000 Gt•km/yr. The geometric mean of these estimates is ≈2,000 Gt•km/yr.

In addition to the regular movement of zooplankton, some 15–50% of zooplankton biomass above 500m is estimated to migrate[185] as part of the diel vertical migration (DVM)[14], with a typical daily distance (round-trip) of ≈1 km/day (the depth difference range was found to be 300-600 m, with an average of ≈400m for both zooplankton and micronekton)[186]. Assuming daily migration of 800 meters for 15-50% of the 10 Gt of mesopelagic zooplankton[12] yields a biomass movement of ≈400-1,500 Gt•km/yr. Using a geometric mean, we estimate the zooplankton migration biomass movement to be ≈800 Gt•km/yr. The zooplankton can vertically move fast relative to their size and hydrodynamic limitations[187] by facilitating unique locomotion mechanisms that are related to buoyancy[14]. Therefore we add this biomass movement to the estimation above, and our best estimate for the total biomass movement of zooplankton is ≈3,000 Gt•km/yr in the pristine and in the modern-day ocean, with a 9-fold uncertainty that is dominated by the uncertainty in biomass.

Epipelagic fish and mammals

The biomass estimates of marine animals is based on the sum of estimates by Hatton et al.[12], and Greenspoon et al.[7] for marine mammals. The uncertainty range of these estimates is calculated as the simple sum of the endpoints of the reported 95% confidence intervals for each animal group. These biomass uncertainties dominate the uncertainty of our biomass movement estimates. The swimming distance of the various marine animals is derived from swimming speeds and activity patterns as described below. The overall uncertainty is derived from the uncertainties in biomass and distance moved treating the two sources of uncertainty as uncorrelated.

Swimming speed in fish generally scales with their body-mass (M) or body length (L). Their optimal speed, where the least amount of energy is used to travel a unit of distance, is taken as their cruising speed (U), which was found to scale as $U \sim L^{0.45}$ for larger animals[181], in the inertial regime, and $U \sim L^{0.79}$ or $U \sim M^{1/6}$ for smaller animals like fish larvae, in the viscous

regime[181,188,189]. However, the swimming speed of mammals and seabirds was found to be roughly constant, with speeds of ≈1-2 m/s[189,190]. A similar range was also found in laboratory experiments across many fish and crustacean zooplankton[191]. These measured values vary about tenfold between different species of fish[191].

The total biomass in the pelagic pristine ocean, before industrial fishing and whaling (before 1850)[12], is roughly uniformly distributed across all swimmers according to their body-mass up to ≈1 ton[12,184]. For example the biomass of all swimmers with body-mass in the range 10-100 grams is similar to that in the range 10-100 kg. Multiplying the biomass of marine animals by their cruising velocity and time of swimming results in their biomass movement. Fish and other marine animals have diverse activity patterns, with some being active throughout the day, and some only during specific times. We assume an average activity of 50% of the time, as most fish in temperate and tropical latitudes are most active during either the day or the night[192], with a 2-fold uncertainty, covering the range from 6 hours per day to all day long. Using allometric relations between body-length and speed described above, and allometric relations between body-mass and body-length[184,193,194], we find the total active biomass movement of epipelagic fish to be ≈70,000 Gt•km/yr in the pristine ocean, and ≈25,000 Gt•km/yr in the current ocean, with a 6-fold uncertainty.

Data-based allometric scaling of the swimming speed of marine mammals[189,190] shows that the average swimming speed is roughly constant per animal body mass, with a relatively narrow distribution. This is supported by theory, which predicts that their speed scales as a power law within the range M^0 - $M^{0.03}$. When applying this roughly constant velocity, of ≈1.3 m/s to swimming mammals,[189,190] according to their estimated pristine biomass[9] we find their biomass movement to be ≈3,000 Gt•km/yr in the pristine ocean, with a 4-fold uncertainty. Applying today's biomass value[7] results in ≈900 Gt•km/yr with a 4-fold uncertainty. If we were to assume the general velocity scaling, as made for fish, we find the biomass movement to double.

The current biomass movement of all marine animals is smaller than the one in the pristine ocean, especially for large animals[9,12]. The total marine biomass is estimated to have been reduced by ≈15% since 1850, but this reduction includes a decline of tens of percent in epipelagic fish, and ≈90 percent decline for marine mammals. The total biomass movement in the ocean is estimated to have been reduced from ≈80,000 Gt•km/yr, to ≈30,000 Gt•km/yr. This reduction was dominated by the loss of large animals.

Supplementary Table 6: Overview of biomass movement in the ocean. Rounded to a single significant digit. The uncertainty refers to both the pristine ocean and the current biomass movement.

	Pristine ocean biomass movement [Gt•km/yr]	Current biomass movement [Gt•km/yr]	Fold uncertainty [×, ÷]
Epipelagic fish	70,000	20,000	6
Mesopelagic fish	7,000	6,000	4
Zooplankton	3,000	3,000	9
Marine mammals	3,000	1,000	4
**Total**	**80,000**	**30,000**	**5**

Mesopelagic fish

Repeating for mesopelagic fish the estimate procedure made for epipelagic fish, assuming viscous swimming, results in a biomass movement of ≈7,000 Gt•km/yr in the pristine ocean, and ≈6,000 Gt•km/yr nowadays with a 4-fold uncertainty. The wet biomass of mesopelagic fish is ≈5 Gt[12] with a threefold uncertainty, and is considered relatively well conserved. Therefore, the total biomass movement estimated is equivalent to assuming constant swimming with a speed of ≈4 cm/s for all mesopelagic fish. This is roughly twice as fast as the measurements reported in [195–197], but agrees with the commonly found velocity range of 0.5-1 body lengths per second, which was also reported in[195–197]. The difference in average speed originates from assuming the biomass spectrum distribution of[12] with respect to fish body-mass. This biomass spectrum is supported by ample evidence and theory[181,184], but shows some variability that we account for within the reported uncertainty.

Prokaryotes

There are about 20-30 GtC of prokaryotes in the world[16,198], most are uncharacterized bacteria in the deep subsurface (terrestrial and oceanic). This is equivalent to less than ≈200 Gt wet mass. Only a fraction of these is motile (can actively move), where the fastest ones have flagella and reach speeds of tens of micrometers per second[199,200]. Archaea and bacteria that have no flagella move much slower, some being sessile[200]. Lack of nutrients and physical characteristics of the surrounding media may significantly reduce this motility[201], which is probably commonplace for bacteria in the deep subsurface. If the average velocity over a full year of all prokaryotes were assumed to be ≈10 micrometers per second, it would result in a total biomass movement of ≈50 Gt•km/yr. This represents an upper bound for prokaryote biomass movement, as only a fraction of the prokaryotes actually moves at any given time, and their active mobility is probably slower.

Sensitivity and uncertainty analysis

One of the most important questions we must address is the estimated uncertainties in all of our results. To that end, this section provides, for the interested reader, a thorough and systematic analysis of these questions. The uncertainties are summarized in Supplementary Table 7 and Fig. 1.

Supplementary Table 7: Summary of the biomass movement estimated uncertainty ranges and upper bounds for all terrestrial animals and humans. Uncertainty ranges assume complete correlation of errors. The total biomass movement of all terrestrial animals is bounded at <700 Gt•km/yr, which is smaller than our best estimate for humans walking and cycling, being ≈800 Gt•km/yr with a range ≈500 - 1600 Gt•km/yr.

Estimated group	Estimated range [Gt•km/yr]
Wild terrestrial mammals excluding bats	10 - 70<br><150
Wild birds (including penguins)	20 - 70<br><130
Terrestrial arthropods	10 - 170<br><300 (<1,000*)
Earthworms	<3
Nematodes	<0.05
Terrestrial gastropods	<10
Amphibians	<10
Reptiles excluding sea turtles	<10
Bats	<50
**Sum of all terrestrial animals**	**<700**
**Sum of all terrestrial animals - with extreme upper bound for land arthropods**	**<1,500**
**Humans**	**3400 - 7000**

*'Extreme upper bound'. See main text.

We derived uncertainty ranges and performed a sensitivity analysis for the results of the study, as detailed below. The biomass movement estimates are usually derived as a product of biomass and the extent of annual movement per taxonomic group, such as species or order. These estimates are prone to possible systematic biases related to the actual measurement, such as the effect of sampling frequency [100,101], where the distance traveled as approximated by the sum of straight-line (polygonal) segments between the measured positions is shorter than the actual path length traveled. There is currently no reliable and widely applicable method to resolve these systematic biases, which are potentially larger than the statistical (random) variability of the data. Therefore, one should remember that obtaining accurate values is about more than statistics.

We deal with these issues by assuming a full correlation of uncertainties when propagating our errors. It significantly increases the uncertainty range compared to treating the errors as being uncorrelated (random). For example, if the biomass movement of species i is estimated as $BM_i \pm \Delta BM_i$, the biomass movement of N species would be calculated as

$$BM_{tot} = \sum_i^N (BM_i \pm \Delta BM_i) \quad (1)$$

such that the overall uncertainty would be

$$\Delta BM_{tot} = \pm \sum_i^N \Delta BM_i \quad (2)$$

instead of the uncertainty due to random errors, given by the variance formula:

$$\sqrt{\sum_i^N (\Delta BM_i)^2}\,. \quad (3)$$

As an example, if for all species $\Delta BM_i = 1$, then $\sum_i^N \Delta BM_i = N$, while $\sqrt{\sum_i^N (\Delta BM_i)^2} = \sqrt{N}$.

Similarly, if a certain animal species is estimated to have a biomass $B_i \pm \Delta B_i$ and to travel a distance $D_i \pm \Delta D_i$, we take their product (the biomass movement) as $(B_i \pm \Delta B_i) \bullet (D_i \pm \Delta D_i)$ rather than $\sqrt{(B_i \Delta D_i)^2 + (D_i \Delta B_i)^2}$. We exemplify the resulting differences using our actual data below.

We additionally performed upper-bound estimates that are more robust to systematic biases and can be made for all groups of animals, including those with insufficient data for more precise estimates. These estimates are made using data or a method different from our regular estimates, and their magnitude exceeds the likely biomass-movement interval. For example, we used measurements of migration speed of birds (km/day) to form an upper estimate, rather than their daily distance traveled. Birds typically move at greater speeds (greater distances per day) when migrating, and move at a path that is much less tortuous (more straight). This results in a larger estimate that is also less prone to the possible bias due to a low sampling frequency.

In the tables below, all uncertainty ranges assume full correlation of errors unless stated otherwise.

In the main text we report all estimates stating only significant digits, thus supplying information on the precision of the reported values[202]. Since our relative uncertainty is often large, when we report our estimates we typically round them to a single significant digit. For example, our best estimate for the total biomass movement of land animals is calculated as about 26.3658 Gt•km/yr with a range of about 9 - 73 Gt•km/yr. Reporting an estimate having several non-zero digits would suggest that our accuracy is very high. In the presented case, it would suggest an accuracy at the 0.00001 Gt•km/yr level. Since our uncertainty and best estimate have the same order of magnitude, we report this value as 30 Gt•km/yr, acknowledging that even the leading digit is uncertain. Similarly, the biomass movement of humans is reported as 4,000 Gt•km/yr instead of the calculated 4,368. We stress that this rounding is made only when reporting the final estimates and not when calculating them.

In this section, we describe the uncertainties while keeping more digits when rounding to increase the transparency of our estimates. We also include below Supplementary Table 8 describing the rounding made in the key reported best estimates that appear in the main text:

Supplementary Table 8: Calculated key estimates and their rounded reported value.

Quantity	Calculated value	Reported value (rounded to one significant digits)
Biomass movement of wild terrestrial mammals excluding bats	26.4 Gt•km/yr	≈30 Gt•km/yr
Biomass movement of wild birds	32.1 Gt•km/yr	≈30 Gt•km/yr
Biomass movement of land arthropods	37.9 Gt•km/yr	≈40 Gt•km/yr
Total human biomass movement	4,368 Gt•km/yr	≈4,000 Gt•km/yr
Total biomass movement in the pristine ocean	81,000 Gt•km/yr	≈80,000 Gt•km/yr
Total biomass movement in the modern ocean	33,700 Gt•km/yr	≈30,000 Gt•km/yr

Wild land mammals excluding bats

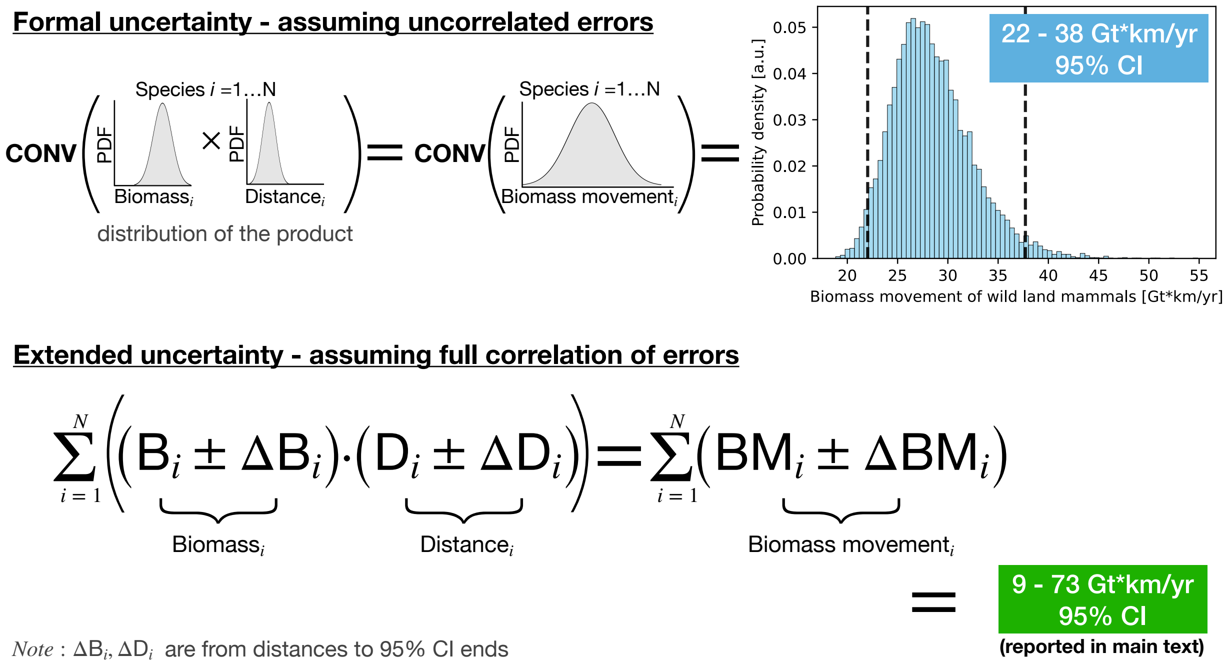

Supplementary Figure 5. Overview of uncertainty estimation for wild land mammals. Top: results of a statistical analysis summing all species-specific uncertainties as uncorrelated errors. Species-specific biomass distributions are based on Greenspoon et al. [7], while species-specific distance distributions are derived from linear regression models that are based on data for 186 species. We used a Monte-Carlo sampling method to produce the distribution of the product of biomass and distance, and to approximate the sum of all species-specific distributions, represented by the convolution (CONV). Dashed lines represent 2.5 and 97.5 percentiles. Bottom: results of an uncertainty analysis that sums the 95% CI bounds of all the species-specific biomass and distance estimated distributions, treating them as completely correlated. The uncertainties are not necessarily symmetric around the mean estimates. This is the uncertainty reported in the main text.

The biomass movement is estimated as the sum of the products of the global biomass of each ≈4000 species by its total annual movement, in the form $\sum_{i \in all\ species} B_i \cdot D_i$, where B_i is the biomass of species *s*, and D_i its total annual distance, as seen in Supplementary Figure 5. For example, the biomass movement of white-tailed deer is about 4.8 Gt•km/yr, being the product of about 2.68 Mt and about 1800 km/yr. The biomass uncertainty is from Greenspoon et. al.[7] and is about 2-fold for each species, with a combined total uncertainty range of 12-38 Mt (excluding bats, which represent ≈7% of the total biomass of wild terrestrial mammals).

The biomass movement and its extended uncertainty is calculated in the following way:

- We acquired data for daily or annual movements of 186 wild land mammalian species [11,87,89–92] that represent about half of the total biomass of wild land mammals.

- Following Carbone et al., we have derived a linear regression model between the logarithm of the body-mass and the logarithm of the measured daily range for all land mammals, and its 95% confidence and 95% prediction intervals.
- Following Carbone et al., we have derived additional linear regression models between body-mass and measured daily range for every taxonomic order that had >4 data points. We also estimated their 95% confidence intervals.
- For each species, we took the daily distance that represents our best estimate based on the following preference: average measured value of total cumulative annual distance, average measured value from Carbone et.al., order-specific extrapolated value, general extrapolated value.
- For the traveled distance uncertainty bounds, we took for each species the max or the min of all the 95% confidence interval values calculated above, and all the measured values.
- The biomass per species and its overall uncertainty range of 12 - 38 Mt is taken from Greenspoon et al.[7] after removing the biomass of bats.
- The reported biomass movement uncertainty range of 9 - 73 Gt•km/yr corresponds to the sum of the products of all the upper or lower biomass bounds by the upper or lower annual traveled distances (see Supplementary Figure 5). This is rounded to 10 - 70 Gt•km/yr when reported in the main text, where the best estimate is rounded from 26.4 Gt•km/yr to 30 Gt•km/yr.

We also produced a second uncertainty range based on a formal statistical analysis under the assumption of uncorrelated errors. We modeled the biomass of each species using the reported 2-fold uncertainty[7] and a log-normal distribution. This slightly increased the uncertainty for some mammals for which there are better population estimates, but did not significantly change the overall estimate. We derived probability distributions for the extrapolated daily movement of each species from the confidence interval of the general linear regression model. We assumed a normal probability distribution of daily movement distance for species with a measured distance. Its standard deviation is assumed to be 25 percent of the measured distances. Using a Monte-Carlo sampling method with 100,000 repetitions, we derived a probability distribution for the combined biomass movement of all species, and extracted its 2.5 and 97.5 percentiles, representing its 95% CI. This resulted in an uncertainty range of 22 - 38 Gt•km/yr. We also sampled the prediction interval of the regression model rather than its confidence interval, which resulted in a similar uncertainty range of 25 - 46 Gt•km/yr.

In addition, we calculated an upper bound estimate of 141 Gt•km/yr for the biomass movement of wild land mammals. It assumed the upper biomass range of Greenspoon et al. [7] for all species. For species with no movement measurements, we took their daily distance as the 97.5% percentile of their prediction interval, representing the 97.5% percentile of the possible range of daily distance as supported by the measurements (rather than the range of the predicted mean daily distances that is calculated from the confidence interval). For mammals with direct distance

measurements, the reported measured distance was taken. This is rounded to 150 Gt•km/yr when reported.

Supplementary Table 9 summarizes the uncertainty range estimated using the different methods, and shows how it changes when including the uncertainty in biomass, in distance traveled, or in both.

Supplementary Table 9: Sensitivity table for biomass movement of wild land mammals. Uncorrelated errors assume errors are random, while correlated errors assume a complete correlation of the errors. The uncertainty range reported in the main text is marked in bold. See Supplementary Figure 5 and supplementary text for more details.

Origin of uncertainty	Biomass movement uncertainty range [Gt•km/yr]
Uncertainty in biomass estimates	Uncorrelated errors: analysis: 23 - 34
	Correlated errors: 15 - 50
Uncertainty in distance traveled estimates	Uncorrelated errors: 23 - 30
	Correlated errors: 15 - 38
Uncertainty in biomass and distance traveled estimates	Uncorrelated errors: 22 - 38
	Correlated errors: **9 - 73**
	Upper bound: 141

Wild birds

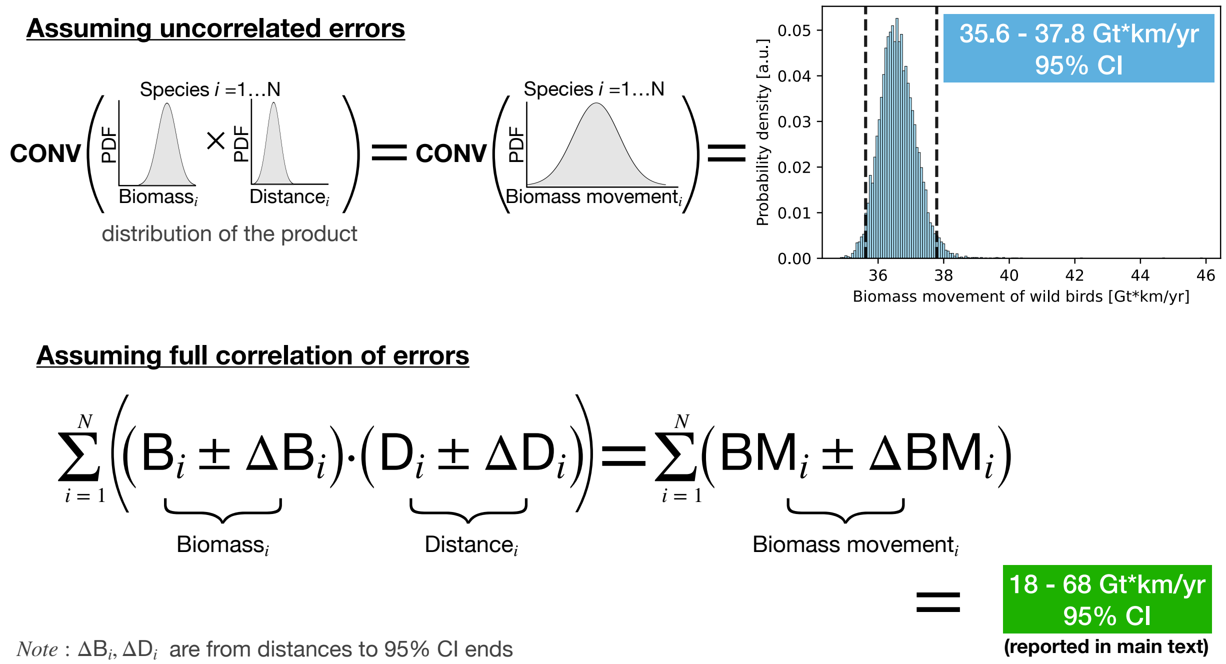

Supplementary Figure 6. Overview of uncertainty estimation for wild birds. Top: results of a statistical analysis summing all species-specific uncertainties as uncorrelated errors. Species-specific biomass distributions are based on Random Forest machine learning regression of upper and lower global population estimates for ≈3,900 bird species (by BirdLife international[94]), as detailed in the supplementary text. Conversion to biomass was made using known species-specific body-mass. Distances were derived from analysis of movement tracks, synthesis of published measurements, and bootstrapping by categories as detailed in the supplementary text. We used a Monte-Carlo sampling method to produce the distribution of the product of biomass and distance, and to approximate the sum of all species-specific distributions, represented by the convolution symbol. Dashed lines represent 2.5 and 97.5 percentiles. Bottom: results of an uncertainty analysis that sums the 95% CI bounds of all the species-specific biomass and distance estimated distributions, treating them as completely correlated. The uncertainties are not necessarily symmetric around the mean estimates. This is the uncertainty reported in the main text.

The biomass movement of birds is estimated as the sum of products of the global biomass of all bird species by its total annual movement $\sum_{i \in all\ species} B_i \cdot D_i$, where B_i is the biomass of species i, and D_i its total annual distance. The full details of the calculation procedure are described under the section ‘Wild birds’ of the supplementary text above.

The biomass estimates are based on global population estimates of 3869 bird species by BirdLife international[94], most of which are given as a range of minimal and maximal population estimate, and some are given as a single figure estimate, where the ‘minimal’ and ‘maximal’ estimates are identical. We converted these population sizes into biomass using adult body-mass data[95]. The total biomass uncertainty range of these 3869 species was calculated by summing all the minimal population estimates and all the maximal population estimates, resulting in 1.8 - 3.1 Mt.

To estimate the biomass of all other birds, we trained a Random Forest machine learning algorithm on a random sample of 90% of the species with known biomass, range, taxonomy, extinction risk, and traits, and repeated it ten times for cross validation. The uncertainty range was found by training the model on only the minimal or only the maximal population estimates, and summing all of them. It resulted in a total biomass estimate of 0.7-2.0 Mt for the birds with extrapolated biomass estimates.

Summing the two estimated ranges assuming a complete correlation of errors yields 2.5-5.1 Mt for all wild birds. Assuming the errors are uncorrelated yields a range of 3.3 - 3.5 Mt.

We also developed an alternative estimate range that is based on naive extrapolation of the known biomass per species. It assigned the average biomass per species using various groupings by taxonomic order, IUCN extinction risk, migration category, and primary lifestyle

We calculated the average minimal or maximal biomass of the 3869 species divided into groups by their IUCN extinction risk category, and by their taxonomic order. These averages were assigned as the minimal or maximal biomass per species to all the remaining species, for which we didn’t have population estimates, according to the groups they belong to. 65 bird species were in groups where no biomass estimates were available, and we assigned them the average of the minimal or maximal biomass per species of their taxonomic order alone. We also made alternative estimates by repeating this procedure while additionaly splitting the groups by the migration category of each species[95], or by excluding the extinction risk category or order in the grouping stage.

We found a total biomass range of 4-12 Mt across the alternative estimates. This range agrees with the range found above, but is higher due to the effect of species with exceptionally large biomass. Removing from this procedure 134 species with exceptionally high biomass (being over 2 standard deviations away from the mean of their extinction risk and order group), representing 1 Mt in total, we find the total biomass to be in the range 4 - 7 Mt when grouping by extinction risk and order, and 3-5 Mt when grouping by taxonomic order alone. This agrees well with the above estimate based on a Random Forest regression, the ≈4 Mt estimate of[97], and the lower estimate of [13].

To get an upper bound estimate for the daily distance birds move, we concentrate on migration speed data of 115 bird species from 35 families of 14 orders[102]. The migration speed of most species was found to be in the range 20–100 km/day, with an average of about 67 km/day. Applying the average migration speed to an upper biomass estimate of 5.1 Mt for all birds results in an upper bound of almost 125 Gt•km/yr.

Supplementary Table 10: Summary of the biomass uncertainty range estimates of wild birds. Uncorrelated errors assume errors are random, while correlated errors assume a complete correlation of the errors. See supplementary text for more details.

Quantity	Biomass uncertainty range [Mt]
Global biomass of 3869 species with estimated global population[94]	Correlated errors:<br>1.8 - 3.1 Mt
Global biomass of species with extrapolated biomass	Correlated errors:<br>0.7 - 2.0 Mt
Combined global biomass of all species	Uncorrelated errors:<br>3.3 - 3.5 Mt<br><br>Correlated errors:<br>**2.5 - 5.1 Mt**
Alternative extended range	3 - 12 Mt

Supplementary Table 11: Summary of the biomass movement uncertainty range estimates of wild birds. Uncorrelated errors assume errors are random, while correlated errors assume a complete correlation of the errors. The uncertainty range reported in the main text is marked in bold. See supplementary text for more details.

Quantity	Biomass movement uncertainty range [Gt•km/yr]
Total biomass movement	Correlated errors:<br>35.6 - 37.8<br><br>Uncorrelated errors:<br>**18 - 68**<br><br>Upper bound:<br>125

Terrestrial arthropods

The biomass movement of terrestrial arthropods is evaluated based on published biomass estimates[23], typical velocities of the various groups, and rough movement characteristics such as movement durations as explained for each group in the supplemental text above and in file 'terrestrial arthropods mobility.xlsx'. Supplementary Table 12 summarizes the uncertainty ranges of our estimates, with the total estimate assuming full correlation of errors. Our upper bound includes a 4 times larger estimate for above-ground arthropods relative to the upper range of their uncertainty range, with a total of ≈300 Gt•km/yr. We also estimate an extreme upper bound for each group, *simultaneously* taking the maximal plausible value of every uncertain quantity in the evaluation of the biomass movement of every land arthropod group: taking their maximal biomass estimates and the maximal estimate for annual distances or the maximal average speed and maximal activity times, resulting is combined estimate of ≈1,000 Gt•km/yr.

To place these extreme upper bounds in perspective, we compare the extreme bound for flying insects, having the highest uncertainty in biomass[23] and highest capacity to move, to the biomass movement of the monarch butterfly (*Danaus plexippus*) - one of the most studied insects and among the most remarkable long-distance insect fliers in the world, crossing the entire United States twice a year as a species.

There are roughly 400,000 known species of Lepidoptera, Diptera, Hymenoptera, Hemiptera, Orthoptera, and Odonata on Earth[208], representing almost all the candidates for long-distance arthropod flyers in the world. We estimate below that the monarch butterfly has a biomass-movement of ≈0.4 Mt•km/yr as the product of ≈50 tons biomass and ≈8,000 km/yr. If we were to assume that all of the 400,000 species mentioned above have an average biomass movement as large as that of the monarch butterfly (which is clearly an overestimate), we would get a total biomass movement of ≈400,000 $\times$ 0.4 Mt•km/yr = 160 Gt•km/yr. This is less than half the extreme upper bound for flying insects in Supplementary Table 12.

Supplementary Table 12: Summary of the biomass movement uncertainty range estimates of land arthropods. The uncertainty range reported in the main text is marked in bold. See supplementary text for more details.

Group	Biomass movement mean estimate [Gt•km/yr]	Biomass movement uncertainty range [Gt•km/yr]	Extreme upper bound [Gt•km/yr]
Termites	12	3 - 54	300
Ants	14	3 - 62	280
Soil microarthropods	0.03	0.002 - 0.6	1
Other soil and plant-litter arthropods	0.3	0.03 - 3	20
above-ground arthropods - non flying	3	1.5 - 6	30
above-ground arthropods - flying	9	2 - 43	370
**All terrestrial arthropods**	**38**	**9 - 169**	**1,000**

Humans
We estimate the total passenger-kilometer of each mode of transportation and each country or economic income group of countries, and multiply it by the average body-mass at each income group to arrive at a biomass movement estimate (see SI text above and file S1 for full details).

The average human body mass was previously estimated at ≈50 kg[13], and we refined this estimate as part of another study[9], based on country-level data for height, BMI, and the age distribution of the population. We found it to be 51.8 - 55.3 kg / person globally, with average estimates of ≈42, 47, 58, and 68 kg for lower-, lower middle-, upper middle-, and higher-income countries, respectively.

Below we summarize the uncertainty ranges of human transportation in units of trillion passenger-kilometers (pkm) per year by mode of transportation, and also present its division into reported and extrapolated data.

For all transportation modes except air travel, we extrapolated a best estimate as well as minimal and maximal estimates for countries without reported data. These extrapolations were based on linear regression models that used GDP per capita as a predictor for pkm per capita, both on a logarithmic scale, as described in the SI text above and in file S1. We also extrapolated the data based on the average traveled distance per capita for each mobility mode and each economic income group. The uncertainty range used the minimum and maximum of the different extrapolations, including their own uncertainties for passenger road mobility. For truck drivers, we have used freight transport data (ton-km) and assumed it to be transported by trucks carrying 5, 10, or 25 tons each, occupying a single driver. The uncertainty range for truck drivers assumes that all extrapolated trucks carry either 5 tons, or 25 tons each. We also added a 10% uncertainty over all the reported data except in air travel, as the published estimates didn't include uncertainties.

For air travel, we have used a range of published estimates[62,63] that covered 96% of the global population and took its extreme values as the uncertainty range.

The combined estimate, assuming complete correlation of errors, is 3404 - 6962 Gt•km/yr, while adding the uncertainties of the different modes of transportation as independent errors results in an uncertainty of ±1206 Gt•km/yr, which we rounded to ±1000 Gt•km/yr when reported above.

Supplementary Table 13: Summary of the biomass movement uncertainty range estimates of humans. See supplementary text for more details.

**Mode of transportation**	**Uncertainty range [trillion pkm/yr]**	**Estimate type**
Passenger road mobility	36 - 59	Total estimate
	27 - 33	Reported data
	9 - 26	Extrapolation
Truck drivers	3 - 7	Total estimate
Rail mobility	3.2 - 4.2	Total estimate
	3.2 - 3.8	Reported data
	0.04 - 0.4	Extrapolation
Walking and cycling	10 - 25	Total estimate
	7 - 9	Reported data
	3 - 16	Extrapolation
Air travel	8 - 10	Total estimate based on reported data
**All modes**	**60 - 105**	

Humans in 1850

We summarize the range of the different estimates for passenger-kilometers in 1850, and its total estimate.

Supplementary Table 14: Summary of the passenger-kilometer estimates for humans in 1850 across transportation modes.

Transportation mode	Passenger-km estimate [billion pkm/yr]	Passenger-km range [billion pkm/yr]
Air travel (balloons)	0	0
Horse carriages	5	2 - 8
Rail	30	10 - 100
Horse riding	100	20 - 500
Walking and running	2,600	1,500 - 3,900
**All modes**	**2,700**	**1,500 - 4,500**

Passive animal movement

We focus our analysis on active animal biomass movement, but it is worth addressing the extent of passive movement as well. Our main estimates include some intentional movement that can be considered passive, such as the gliding of birds or vertical movement of zooplankton via buoyancy. As for the passive biomass movement of other animals, there is currently not enough data to estimate it with certainty, but we can estimate an upper bound for it over land. Passive movement includes movements powered by wind, other animals, water, and humans.

Other than birds, wind-driven passive movement typically mostly affects small animals such as flying insects, ballooning spiders, and micro-arthropods. It is therefore likely that high-flying insects constitute a large fraction of this wind-driven passive animal movement. The biomass of such high-flying insects was estimated[23] to have an upper bound of a few megatons dry weight, or several megatons wet weight. Assuming as an upper bound that all of this biomass moves 1,000 km a year on average, similar to the longest insect mass migrations[123], their biomass movement contribution is bounded at several Gt•km/yr.

Passive movement via other animals includes phoresy[203] and movement of parasites via their hosts. Since the mass of the transported animal (the phoront or parasite) is typically much smaller than that of the host animal, we can safely assume that the biomass movement of such movement is much smaller than that of the host animals, and we can include such movements within the uncertainty range of the biomass movement of the host animals.

Passive movement by ocean currents, rivers, waves, and other water movements could account for a significant biomass movement of aquatic animals, despite recent studies finding it to be more limited than previously thought (e.g.[204,205] and references therein). This is especially significant in the context of planktonic animals, which include a large fraction of the global animal biomass[12,13]. Mapping this movement is beyond the scope of the current study, and should be addressed in future studies.

Animal transport by humans is dominated by the transport of livestock, which is covered in the biomass movement estimate of livestock. Transport of live fish and other live aquaculture could also be significant, with a total production of about 200 Mt/yr[206]. We are not aware of an estimate regarding how much of this biomass is being transported live. We can very crudely assume that all farmed animals (≈100 Mt/yr) are being transported on average less than 100 km while being alive, and ≈ten percent of the other aquaculture move less than 1,000 km while alive, giving a total of less than 20•Gt km/yr. Animal transport by humans also includes transport of other live animals such as pets, and unintentional animal transport, such as transport of invasive species by trade (e.g.[207,208] and references therein). To get an upper bound for the this biomass movement, we assume that such unintentional animals within traded goods correspond to less than 10^{-4}% of the total transported food biomass of ≈8,000 Gt•km/yr[17], corresponding to a severe

infestation (being roughly the mass ratio of ten fruits such as apples to an ant), resulting in an upper bound of 8 Mt•km/yr. In addition, ballast water is a significant source for marine animal transport, with ≈10 Gt of ballast water discharge per year[209]. To get an upper bound for the amount of transported animal biomass in ballast water, we assume that all the ≈10 Gt of marine animals are spread evenly in the upper ten meters of the oceans accessible to marine vessels, with an area of about 360 million square kilometers. The marine animal biomass accounts for ≈10^{-4}% of the weight of this water, hence the ≈10 Gt/yr of ballast water can hold up to ≈10 kt of animals. Assuming this water moves on average less than 10,000 km, the total biomass movement of these marine animals is bounded by 100 Mt•km/yr (0.1 Gt•km/yr).

The passive movement of land animals is thus much smaller than the thousands of Gt•km/yr by humans. However, the significance of such passive movements calls to fill the current knowledge gaps, and to better quantify the passive movement of animals in the biosphere.

Migrations, gatherings and commutes

Fig. 2 of the main text is based on the following estimates.

Monarch butterflies

About 100 million Monarch butterflies (*Danaus plexippus*) migrate annually from North America to overwintering grounds in Mexico and back[125]. The eastern population covers migration distances of ≈6,000 to ≈9,000 km round trip (based on rough estimate of migration map in[125], and references therein). This represents direct flight distances, and actual total path lengths might be longer. Each adult butterfly weighs ≈0.5g[210], giving a total of ≈50 tons of wet biomass. Assuming total traveling distance of ≈8,000 km gives an estimate of 0.4 Mt•km/yr.

Arctic terns

Arctic terns complete annual migration circuits of about 50,000-70,000 km between the breeding areas in the northern coasts/tundra, and the Antarctic pack-ice zone[211]. Their global population is estimated at ≈2 million individuals[212], with body weight of about 110 grams[95]. This gives a total biomass movement of ≈16 Mt•km/yr.

Gray wolves

There are ≈200,000 gray wolves globally, with an adult body mass of ≈30 kg[7]. They travel on average ≈14 km/day[11,213,214], having a total biomass movement of ≈30 Mt•km/yr.

Serengeti migration

About 1.3 million gnus participate in the Serengeti wildebeests migration, covering an estimated distance of ≈2000-3000 km/yr[11,215]. Blue wildebeest (*Connochaetes taurinus*) adult body mass is

≈200 kg[7], thus their total biomass movement in the Serengeti is ≈700 Mt•km/yr. The wildebeest are accompanied by ≈200,000 plains zebras (*Equus burchelli*)[216,217] with adult body mass of ≈200 kg[7], adding up to ≈100 Mt•km/yr to the migration. A similar number of Thomson's gazelle (*Eudorcas thomsonii*)[218] and Grant's gazelle (*Nanger granti*)[219] also migrate in the Serengeti ecosystem, but their body mass is only several tens of kilograms[7], hence their biomass movement is small compared to that of the wildebeests and zebras. The total biomass movement of the Serengeti migration is thus ≈800 Mt•km/yr.

Humpback whales

Humpback whales travel between reproductive areas in tropical regions and high latitude feeding grounds. The humpback population is divided into several sub-populations in both hemispheres, where their annual migrations are typically in the range of ≈12,000-17,000 km per year[220–222]. Its population has ≈84,000 mature individuals out of ≈135,000 whales altogether. An adult humpback whale has a mass of ≈30 metric tons[7]. Assuming an average distance of ≈15,000 km/yr for the adult population gives ≈40 Gt•km/yr of biomass movement.

Diel vertical migration (DVM)

Global estimates[223] of the daily vertical movements of mesopelagic fish using sonar backscattering at 38 kHz have found that ≈50% of mesopelagic fish travel vertically towards the surface ≈400 m for a total of ≈800 m every day. Assuming that half of them travel 800 meters daily results in a yearly biomass movement of ≈700 Gt•km/yr, with a 4-fold uncertainty. This movement is part of the diel vertical migration and is included in the estimate of mesopelagic fish swimming described above. Zooplankton migration biomass movement was estimated above to be ≈800 Gt•km/yr, giving a combined biomass movement of ≈1,500 Gt•km/yr.

Hajj

The Hajj Islamic pilgrimage to Mecca, Saudi Arabia, included the participation of ≈2-3 million pilgrims annually prior to the COVID pandemic. El Hanandeh[224] estimated the total human mobility in Hajj in the year 2011 while estimating its carbon footprint. He found that ≈1.5 million international pilgrims came by air, traveling in total ≈12 billion pkm, and another ≈1 million pilgrims arrived by road, traveling ≈2.4 billion pkm in total. In addition ≈1.7 billion pkm were traveled domestically by road as part of the pilgrimage. Assuming that the pilgrims are adults, having an average body weight of 65 kg per person, this corresponds to ≈1 Gt•km per 2011 pilgrimage. See file S4 for the full calculation.

World cup

About a million international spectators arrived at Qatar for the 2022 FIFA world cup, traveling ≈13,000 km return trip[225] on average, accounting for ≈0.8 Gt•km/yr.

Energy estimates
The energy estimates for animal groups are based on each species' fitted total optimal cost of transport (COT)[21], given by its mode of locomotion and body mass. Multiplying the biomass movement by the COT results in the total power used. The uncertainty in the total power used by each species is composed by the uncertainty in its biomass movement, and the uncertainty in the COT. We crudely assume that the uncertainty in the COT across all animal groups is 2-fold.

Wild mammals
Using the general dependance of the COT of terrestrial mammals on their body-mass[21], we found the total average power used by all terrestrial mammals to be ≈2 GW, with a biomass-averaged COT of ≈3 J/kg•m and a 3-fold uncertainty.

Wild birds
Using the general dependance of the COT of actively flying animals on their body-mass[21], and applying the COT of swimming for penguins, and the COT of walking for terrestrial birds, we found the total average power used by all wild birds to be ≈9 GW (3-fold uncertainty), with a biomass-averaged COT of ≈9 J/kg•m. This is perhaps an overestimate, as birds often use air currents to travel large distances, reducing their cost of transport.

As a comparison, Nyffeler et.al.[97] have estimated that ≈3 Mt of insectivorous birds consume ≈400 Mt of prey each year, with an energy content of $\approx 2.7 \times 10^{18}$ J per year (750 TWh/year). This is equivalent to ≈85 GW, or about five times more than the energy of mobility that we estimated.

Based on ≈8.5 trillion global revenue pkm (RPK) estimated by ICAO in 2019[62], consuming ≈8.5 EJ[22], results in ≈1 MJ/RPK. The largest airline carriers deliver ≈300 billion RPK per year[226], consuming on average ≈10 GW.

Marine animals
The biomass movement of marine mammals was estimated at ≈900 Gt•km/yr with a 4-fold uncertainty. Estimating their COT from their body-mass[7,21] results in a biomass-averaged COT of ≈0.8 J/kg•m, and a total average power of ≈20 GW (4-fold uncertainty).

Global Maritime transport consumes ≈11 EJ/yr[22], transporting freight of ≈110,000 Gt•km/yr[18], with natural gas and chemicals being transported ≈7,500 Gt•km/yr, or ≈7% of the total. This corresponds to ≈24 GW average power (≈10 GW for chemicals and ≈14 GW for natural gas). Similarly, oil maritime transport consumes ≈80 GW.

Similar to the above estimates, we find that all fish spend on average ≈4,000 GW (3-fold uncertainty), which is similar to all human transportation[22], or ≈30% of the final energy use of humanity[22].

References

1. Miller, H. J., Dodge, S., Miller, J. & Bohrer, G. Towards an Integrated Science of Movement: Converging Research on Animal Movement Ecology and Human Mobility Science. Int. J. Geogr. Inf. Sci. **33**, 855–876 (2019).
2. Alerstam, T. & Bäckman, J. Ecology of animal migration. Curr. Biol. **28**, R968–R972 (2018).
3. Ellis-Soto, D. et al. A vision for incorporating human mobility in the study of human–wildlife interactions. Nature Ecology & Evolution **7**, 1362–1372 (2023).
4. Bauer, S. & Hoye, B. J. Migratory animals couple biodiversity and ecosystem functioning worldwide. Science **344**, 1242552 (2014).
5. Demšar, U. et al. Establishing the integrated science of movement: bringing together concepts and methods from animal and human movement analysis. Int. J. Geogr. Inf. Sci. **35**, 1273–1308 (2021).
6. Nathan, R. et al. Big-data approaches lead to an increased understanding of the ecology of animal movement. Science **375**, eabg1780 (2022).
7. Greenspoon, L. et al. The global biomass of wild mammals. Proc. Natl. Acad. Sci. U. S. A. **120**, e2204892120 (2023).
8. Hansson, L. A. & Åkesson, S. Animal Movement Across Scales. (Oxford University Press, 2014).
9. Greenspoon, L. et al. The Global Biomass of Mammals Since 1850. In print. DOI: 10.1038/s41467-025-63888-z
10. World Bank. World bank country and lending groups – world bank data help desk. https://datahelpdesk.worldbank.org/knowledgebase/articles/906519-world-bank-country-and-lending-groups. (accessed 01 Mar 2024)
11. Joly, K. et al. Longest terrestrial migrations and movements around the world. Sci. Rep. **9**, 15333 (2019).
12. Hatton, I. A., Heneghan, R. F., Bar-On, Y. M. & Galbraith, E. D. The global ocean size spectrum from bacteria to whales. Sci Adv **7**, eabh3732 (2021).
13. Bar-On, Y. M., Phillips, R. & Milo, R. The biomass distribution on Earth. Proc. Natl. Acad. Sci. U.S.A **115**, 6506–6511 (2018).
14. Bandara, K., Varpe, Ø., Wijewardene, L., Tverberg, V. & Eiane, K. Two hundred years of zooplankton vertical migration research. Biol. Rev. Camb. Philos. Soc. **96**, 1547–1589 (2021).
15. Barnosky, A. D. Colloquium paper: Megafauna biomass tradeoff as a driver of Quaternary and future extinctions. Proc. Natl. Acad. Sci. U. S. A. **105 Suppl 1**, 11543–11548 (2008).
16. Bar-On, Y. M. & Milo, R. Towards a quantitative view of the global ubiquity of biofilms. Nat. Rev. Microbiol. **17**, 199–200 (2019).
17. Li, M. et al. Global food-miles account for nearly 20% of total food-systems emissions. Nat Food **3**, 445–453 (2022).

18. UNCTAD & United Nations Conference on Trade and Development. Review of Maritime Transport 2022: Navigating Stormy Waters. (UN, 2022).

19. Schmidt-Nielsen, K. Locomotion: energy cost of swimming, flying, and running. Science **177**, 222–228 (1972).

20. di Prampero, P. E. The energy cost of human locomotion on land and in water. Int. J. Sports Med. **7**, 55–72 (1986).

21. Williams, T. M. The evolution of cost efficient swimming in marine mammals: limits to energetic optimization. Philos. Trans. R. Soc. Lond. B Biol. Sci. **354**, 193–201 (1999).

22. IEA. Key World Energy Statistics 2021. https://doi.org/10.1787/2ef8cebc-en (2021) doi:10.1787/2ef8cebc-en.

23. Rosenberg, Y. et al. The global biomass and number of terrestrial arthropods. Sci Adv **9**, eabq4049 (2023).

24. Wilson, R. P. et al. Estimates for energy expenditure in free-living animals using acceleration proxies: A reappraisal. J. Anim. Ecol. **89**, 161–172 (2020).

25. Schmitz, O. J. et al. Animals and the zoogeochemistry of the carbon cycle. Science **362**, (2018).

26. Doughty, C. E. et al. Global nutrient transport in a world of giants. Proc. Natl. Acad. Sci. U. S. A. **113**, 868–873 (2016).

27. CMS. State of the world's Migratory Species. https://www.cms.int/en/publication/state-worlds-migratory-species, (accessed 05 Mar 2024).

28. Brennan, A. et al. Functional connectivity of the world's protected areas. Science **376**, 1101–1104 (2022).

29. Tucker, M. A. et al. Moving in the Anthropocene: Global reductions in terrestrial mammalian movements. Science **359**, 466–469 (2018).

30. Doherty, T. S., Hays, G. C. & Driscoll, D. A. Human disturbance causes widespread disruption of animal movement. Nat Ecol Evol **5**, 513–519 (2021).

31. McCauley, D. J. et al. Marine defaunation: animal loss in the global ocean. Science **347**, 1255641 (2015).

32. Dirzo, R. et al. Defaunation in the Anthropocene. Science **345**, 401–406 (2014).

33. Vitousek, P. M., Mooney, H. A., Lubchenco, J. & Melillo, J. M. Human domination of earth's ecosystems. Science **277**, 494–499 (1997).

34. Zalasiewicz, J., Waters, C. N., Williams, M. & Summerhayes, C. P. The Anthropocene as a Geological Time Unit: A Guide to the Scientific Evidence and Current Debate. (Cambridge University Press, 2019).

35. Richardson, K. et al. Earth beyond six of nine planetary boundaries. Sci Adv **9**, eadh2458 (2023).

36. Elhacham, E., Ben-Uri, L., Grozovski, J., Bar-On, Y. M. & Milo, R. Global human-made mass exceeds all living biomass. Nature **588**, 442–444 (2020).

37. Rosenberg, Y. *et al*. Human biomass movement exceeds the biomass movement of all land animals combined [Data set]. *Zenodo.* 10.5281/zenodo.16731771 (2025)

38. European Commission, Directorate-General for Mobility and Transport. EU Transport in figures. https://op.europa.eu/s/z768, (accessed 01 Mar 2024).

39. World Bank. Railways, passengers carried (million passenger-km). World Bank Open Data

https://data.worldbank.org/indicator/IS.RRS.PASG.KM. (accessed 01 Mar 2024).

40. Virág, D. et al. How much infrastructure is required to support decent mobility for all? An exploratory assessment. Ecol. Econ. **200**, 107511 (2022).
41. Tomschy, R. et al. Österreich unterwegs 2013/2014. Ergebnisbericht zur österreichweiten Mobilitätserhebung Österreich unterwegs: Wien, Austria (2016).
42. Barban, P., De Nazelle, A., Chatelin, S., Quirion, P. & Jean, K. Assessing the Health Benefits of Physical Activity Due to Active Commuting in a French Energy Transition Scenario. Int. J. Public Health **67**, 1605012 (2022).
43. UK Department for Transport. National travel survey 2021: Active travel. GOV.UK https://www.gov.uk/government/statistics/national-travel-survey-2021/national-travel-survey-2021-active-travel. (accessed 01 Mar 2024).
44. Vallée, J., Ecke, L., Chlond, B. & Vortisch, P. Deutsches Mobilitätspanel (MOP)--Wissenschaftliche Begleitung und Auswertungen Bericht 2021/2022: Alltagsmobilität und Fahrleistung. Karlsruher Institut für Technologie (KIT): Karlsruhe, Germany (2022).
45. Traficom. Henkilöliikennetutkimus 2021: Suomalaisten liikkuminen. https://www.traficom.fi/fi/julkaisut/henkiloliikennetutkimus-2021-suomalaisten-liikkuminen. (accessed 01 Mar 2024).
46. Centraal Bureau voor de Statistiek. Onderweg in Nederland (ODiN) 2022 - Onderzoeksbeschrijving. Centraal Bureau voor de Statistiek https://www.cbs.nl/nl-nl/longread/rapportages/2023/onderweg-in-nederland--odin---2022-onderzoeksbeschrijving (2023). (accessed 01 Mar 2024).
47. Bundesamt für Statistik. Verkehrsverhalten der Bevölkerung, Synthesetabellen. Bundesamt für Statistik https://www.bfs.admin.ch/bfs/de/home/statistiken/mobilitaet-verkehr/personenverkehr/verkehrsverhalten/tabellen-2021.assetdetail.24267703.html (2023). (accessed 01 Mar 2024).
48. Pakistan Bureau of Statistics. Time Use Survey 2007. Government of Pakistan Preprint at (2009).
49. Luis de Moraes Ferrari, G. et al. Original research Socio-demographic patterning of self-reported physical activity and sitting time in Latin American countries: findings from ELANS. BMC Public Health **19**, 1723 (2019).
50. Trafik analys. Metodrapport RVU Sverige 2011. https://www.trafa.se/transportmonster/RVU-Sverige/metodrapport-rvu-sverige-2011-6329/. (accessed 01 Mar 2024).
51. Zahnow, R., Kimpton, A., Corcoran, J. & Mielke, G. Neighbourhood correlates of average population walking: using aggregated, anonymised mobile phone data to identify where people walk. Health Place **77**, 102892 (2022).
52. Environment, U. N. Walking and cycling in Africa - evidence and good practice to inspire action. UNEP - UN Environment Programme https://www.unep.org/resources/report/walking-and-cycling-africa-evidence-and-good-practice-inspire-action (2022).
53. ISFORT. 16° Rapporto sulla mobilità degli italiani 'Audimob'. https://www.isfort.it/progetti/16-rapporto-sulla-mobilita-degli-italiani-audimob/. (accessed 01 Mar 2024).
54. World Bank. Railways in developing countries: A global review. Mobility and Transport Connectivity Series (2022).
55. Directorate-General for Mobility and Transport, European commission. EU Transport in Figures: Statistical Pocketbook 2020. (Publications Office of the European Union, 2020).
56. Liu, J.-L., Li, M.-Y., Zeng, Y., Yin, M.-J. & Zhang, X.-X. An improved methodology for evaluating

energy service demand for China's passenger transport sector. Advances in Climate Change Research **13**, 290–300 (2022).

57. Jha, A. P. & Singh, S. K. Future mobility in India from a changing energy mix perspective. Econ. Anal. Policy **73**, 706–724 (2022).
58. Tsamboulas, D. & Moraiti, P. Identification of potential target locations and attractiveness assessment due to terrorism in the freight transport. J. Transp. Secur. **1**, 189–207 (2008).
59. World Bank. Railways in developing countries : A global review. World Bank http://documents.worldbank.org/curated/en/099515004292230157/P1766680d0330a0a10ba540e118a9f4da59. (accessed 01 Mar 2024).
60. Finance Division, Ministry of Finance. Bangladesh Economic Review. https://mof.portal.gov.bd/site/page/28ba57f5-59ff-4426-970a-bf014242179e/Bangladesh-Economic-Review-2023. (accessed 01 Mar 2024).
61. Airlines For America. World airlines traffic and capacity. https://www.airlines.org/dataset/world-airlines-traffic-and-capacity. (accessed 01 Mar 2024).
62. ICAO. The World of Air Transport in 2019— Presentation of 2019 Air Transport statistical results. https://www.icao.int/annual-report-2019/Pages/the-world-of-air-transport-in-2019-statistical-results.aspx. (accessed 01 Mar 2024).
63. IATA Sustainability & Economics. Air Passenger Market Analysis December 2023. https://www.iata.org/en/iata-repository/publications/economic-reports/air-passenger-market-analysis-december-2023/ (2024).
64. Our World in Data. Per capita passenger kilometers from air travel. https://ourworldindata.org/grapher/per-capita-km-aviation?tab=table. (accessed 01 Mar 2024).
65. Tudor-Locke, C. et al. How many steps/day are enough? For adults. Int. J. Behav. Nutr. Phys. Act. **8**, 79 (2011).
66. Barreira, T. V. et al. Normative steps/day and peak cadence values for united states children and adolescents: National Health and Nutrition Examination Survey 2005-2006. J. Pediatr. **166**, 139–143 (2015).
67. Althoff, T. et al. Large-scale physical activity data reveal worldwide activity inequality. Nature **547**, 336–339 (2017).
68. Bagwell, P. S. The Transport Revolution from 1770. (Batsford, 1974).
69. Robert C. Feenstra, Robert Inklaar, Marcel Timmer, Pieter Woltjer. Penn World Table 10.0. Groningen Growth and Development Centre https://doi.org/10.15141/S5Q94M (2021).
70. Passenger rail usage. https://dataportal.orr.gov.uk/statistics/usage/passenger-rail-usage/.
71. Derry, M. E. Horses in Society: A Story of Animal Breeding and Marketing, 1800-1920. (University of Toronto Press, 2006).
72. Schaschl, E. Rekonstruktion der Arbeitszeit in der Landwirtschaft im 19. Jahrhundert am Beispiel von Theyern in Niederösterreich. (Wien: Institut für Soziale Ökologie. (Social Ecology Working Papers, 96, Vienna).).
73. Klein Goldewijk, K., Beusen, A. & Janssen, P. Long-term dynamic modeling of global population and built-up area in a spatially explicit way: HYDE 3.1. Holocene **20**, 565–573 (2010).
74. Fischer-Kowalski, M., Krausmann, F. & Pallua, I. A sociometabolic reading of the Anthropocene: Modes of subsistence, population size and human impact on Earth. The Anthropocene Review **1**, 8–

33 (2014).

75. Fajzel, W. et al. The global human day. Proc. Natl. Acad. Sci. U. S. A. **120**, e2219564120 (2023).

76. Knoblauch, R. L., Pietrucha, M. T. & Nitzburg, M. Field Studies of Pedestrian Walking Speed and Start-Up Time. Transp. Res. Rec. **1538**, 27–38 (1996).

77. Gates, T. J., Noyce, D. A., Bill, A. R. & Van Ee, N. Recommended Walking Speeds for Timing of Pedestrian Clearance Intervals Based on Characteristics of the Pedestrian Population. Transp. Res. Rec. **1982**, 38–47 (2006).

78. Bohannon, R. W. & Williams Andrews, A. Normal walking speed: a descriptive meta-analysis. Physiotherapy **97**, 182–189 (2011).

79. IEA. Passenger car fleet and share of SUVs in total car emissions, 2010-2022. https://www.iea.org/data-and-statistics/charts/passenger-car-fleet-and-share-of-suvs-in-total-car-emissions-2010-2022. (accessed 01 Mar 2024).

80. Global Fuel Economy Initiative. Trends in the global vehicle fleet 2023. https://www.globalfueleconomy.org/data-and-research/publications/trends-in-the-global-vehicle-fleet-2023. (accessed 01 Mar 2024).

81. IEA. Global Fuel Economy Initiative 2021. https://www.iea.org/reports/global-fuel-economy-initiative-2021. (accessed 01 Mar 2024).

82. Global Mobility Report: Measuring progress toward safe, clean, efficient, and inclusive transport. World Bank https://www.worldbank.org/en/results/2020/11/11/global-mobility-report-measuring-progress-toward-safe-clean-efficient-and-inclusive-transport (2020).

83. FAA. Benefit-Cost Analysis. https://www.faa.gov/regulations_policies/policy_guidance/benefit_cost. (accessed 01 Mar 2024).

84. Fafard, A. & Kingsley-Jones, M. 2020 airliner census highlights coronavirus's unprecedented impact on fleets. Flight Global https://www.flightglobal.com/air-transport/2020-airliner-census-highlights-coronaviruss-unprecedented-impact-on-fleets/140046.article (2020).

85. OECD. Transport, S. G. Transport - Freight transport - OECD Data. https://data.oecd.org/transport/freight-transport.htm. (accessed 01 Mar 2024).

86. Our World in Data. Tonne-kilometers of air freight. https://ourworldindata.org/grapher/air-transport-freight-ton-km. (accessed 01 Mar 2024).

87. Carbone, C., Cowlishaw, G., Isaac, N. J. B. & Rowcliffe, J. M. How far do animals go? Determinants of day range in mammals. Am. Nat. **165**, 290–297 (2005).

88. Garland, T. Scaling the Ecological Cost of Transport to Body Mass in Terrestrial Mammals. Am. Nat. **121**, 571–587 (1983).

89. Leggett, K. Daily and hourly movement of male desert-dwelling elephants. Afr. J. Ecol. **48**, 197–205 (2010).

90. Webb, S. L., Gee, K. L., Strickland, B. K., Demarais, S. & DeYoung, R. W. Measuring Fine-Scale White-Tailed Deer Movements and Environmental Influences Using GPS Collars. International Journal of Ecology **2010**, (2010).

91. Munn, A. J., Dawson, T. J., McLeod, S. R., Dennis, T. & Maloney, S. K. Energy, water and space use by free-living red kangaroos Macropus rufus and domestic sheep Ovis aries in an Australian rangeland. J. Comp. Physiol. B **183**, 843–858 (2013).

92. Jakopak, R. P. et al. Diel timing of migration is not plastic in a migratory ungulate. Anim. Behav.

192, 51–62 (2022).

93. Hawks, J., Hunley, K., Lee, S. H. & Wolpoff, M. Population bottlenecks and Pleistocene human evolution. Mol. Biol. Evol. **17**, 2–22 (2000).

94. Robinson, O. J. et al. Extreme uncertainty and unquantifiable bias do not inform population sizes. Proc. Natl. Acad. Sci. U. S. A. **119**, e2113862119 (2022).

95. Tobias, J. A. et al. AVONET: morphological, ecological and geographical data for all birds. Ecol. Lett. **25**, 581–597 (2022).

96. Kays, R. et al. The Movebank system for studying global animal movement and demography. Methods Ecol. Evol. **13**, 419–431 (2022).

97. Nyffeler, M., Şekercioğlu, Ç. H. & Whelan, C. J. Insectivorous birds consume an estimated 400–500 million tons of prey annually. The Science of Nature **105**, 47 (2018).

98. Jetz, W., Thomas, G. H., Joy, J. B., Hartmann, K. & Mooers, A. O. The global diversity of birds in space and time. Nature **491**, 444–448 (2012).

99. Cooper, N. W. & Marra, P. P. Hidden Long-Distance Movements by a Migratory Bird. Curr. Biol. **30**, 4112 (2020).

100. Marcus Rowcliffe, J., Carbone, C. & Kays, R. Bias in estimating animal travel distance: the effect of sampling frequency. Methods Ecol. Evol. (2012) doi:10.1111/j.2041-210X.2012.00197.x.

101. Noonan, M. J. et al. Scale-insensitive estimation of speed and distance traveled from animal tracking data. Mov Ecol **7**, 35 (2019).

102. Payevsky, V. A. Speed of bird migratory movements as an adaptive behavior. Biology Bulletin Reviews **3**, 219–231 (2013).

103. Wang, C., Henderson, G., Gautam, B. K. & Chen, X. Lethal and sublethal effects of lufenuron on the Formosan subterranean termite (Isoptera: Rhinotermitidae). J. Econ. Entomol. **107**, 1573–1581 (2014).

104. Yuan, Z. & Ping Hu, X. Evaluation of differential antitermitic activities of Lantana camara oven-dried tissues against Reticulitermes virginicus (Isoptera: Rhinotermitidae). Insect Sci. **18**, 671–681 (2011).

105. do Sacramento, J. J. M. et al. Soldiers of the termite Nasutitermes corniger (Termitidae: Nasutitermitinae) increase the ability to exploit food resources. Behav. Processes **181**, 104272 (2020).

106. Gautam, B. K., Henderson, G. & Wang, C. Localized treatments using commercial dust and liquid formulations of fipronil against Coptotermes formosanus (Isoptera: Rhinotermitidae) in the laboratory. Insect Sci. **21**, 174–180 (2014).

107. Lee, S.-B., Chouvenc, T., Mizumoto, N., Mullins, A. & Su, N.-Y. Age-based spatial distribution of workers is resilient to worker loss in a subterranean termite. Sci. Rep. **12**, 7837 (2022).

108. Hurlbert, A. H., Ballantyne, F., IV & Powell, S. Shaking a leg and hot to trot: the effects of body size and temperature on running speed in ants. Ecol. Entomol. **33**, 144–154 (2008).

109. Schultheiss, P. et al. The abundance, biomass, and distribution of ants on Earth. Proc. Natl. Acad. Sci. U. S. A. **119**, e2201550119 (2022).

110. Food and Agriculture Organization of the United Nations, Global Soil Biodiversity Initiative, Secretariat of the Convention of Biological, European Commission & Intergovernmental Technical Panel on Soils. State of Knowledge of Soil Biodiversity - Status, Challenges and Potentialities:

Report 2020. (Food & Agriculture Org., 2020).

111. Ojala, R. & Huhta, V. Dispersal of microarthropods in forest soil. Pedobiologia **45**, 443–450 (2001).

112. Lehmitz, R., Russell, D., Hohberg, K., Christian, A. & Xylander, W. E. R. Active dispersal of oribatid mites into young soils. Appl. Soil Ecol. **55**, 10–19 (2012).

113. Weisstein, E. W. Random Walk--2-Dimensional. https://mathworld.wolfram.com/RandomWalk2-Dimensional.html. (accessed 13 Jan 2024).

114. Hirt, M. R., Jetz, W., Rall, B. C. & Brose, U. A general scaling law reveals why the largest animals are not the fastest. Nat Ecol Evol **1**, 1116–1122 (2017).

115. Růžičková, J. & Elek, Z. Beetles on the move: Not-just-a-technical review of beetles' radio-tracking. Entomol. Exp. Appl. **171**, 82–93 (2023).

116. Růžičková, J. & Veselý, M. Using radio telemetry to track ground beetles: Movement of Carabus ullrichii. Biologia **71**, 924–930 (2016).

117. Baars, M. A. Patterns of movement of radioactive carabid beetles. Oecologia **44**, 125–140 (1979).

118. Watts, C. & Thornburrow, D. Habitat use, behavior and movement patterns of a threatened New Zealand giant weta, Deinacrida heteracantha (Anostostomatidae: Orthoptera). J. Orthoptera Res. **20**, 127–135 (2011).

119. Kindvall, O. Dispersal in a metapopulation of the bush cricket, Metrioptera bicolor (Orthoptera: Tettigoniidae). J. Anim. Ecol. **68**, 172–185 (1999).

120. Nuhlíčková, S., Svetlík, J., Kaňuch, P., Krištín, A. & Jarčuška, B. Movement patterns of the endemic flightless bush-cricket, Isophya beybienkoi. J. Insect Conserv. **28**, 141–150 (2024).

121. Matenaar, D., Bröder, L., Bazelet, C. S. & Hochkirch, A. Persisting in a windy habitat: population ecology and behavioral adaptations of two endemic grasshopper species in the Cape region (South Africa). J. Insect Conserv. **18**, 447–456 (2014).

122. Joern, A. Small-Scale Displacements of Grasshoppers (Orthoptera: Acrididae) within Arid Grasslands. J. Kans. Entomol. Soc. **56**, 131–139 (1983).

123. Warrant, E. et al. The Australian Bogong Moth Agrotis infusa: A Long-Distance Nocturnal Navigator. Front. Behav. Neurosci. **10**, 77 (2016).

124. Minter, M. et al. The tethered flight technique as a tool for studying life-history strategies associated with migration in insects. Ecol. Entomol. **43**, 397–411 (2018).

125. Reppert, S. M. & de Roode, J. C. Demystifying Monarch Butterfly Migration. Curr. Biol. **28**, R1009–R1022 (2018).

126. Chowdhury, S., Fuller, R. A., Dingle, H., Chapman, J. W. & Zalucki, M. P. Migration in butterflies: a global overview. Biol. Rev. Camb. Philos. Soc. **96**, 1462–1483 (2021).

127. Alerstam, T. et al. Convergent patterns of long-distance nocturnal migration in noctuid moths and passerine birds. Proc. Biol. Sci. **278**, 3074–3080 (2011).

128. Knight, S. M., Pitman, G. M., Flockhart, D. T. T. & Norris, D. R. Radio-tracking reveals how wind and temperature influence the pace of daytime insect migration. Biol. Lett. **15**, 20190327 (2019).

129. Moore, T. C. & Brown, H. E. Estimating Aedes aegypti (Diptera: Culicidae) Flight Distance: Meta-Data Analysis. J. Med. Entomol. **59**, 1164–1170 (2022).

130. Service, M. W. Mosquito (Diptera: Culicidae) Dispersal—The Long and Short of it. J. Med. Entomol. **34**, 579–588 (1997).

131. Dominiak, B. C. Review of Dispersal, Survival, and Establishment of Bactrocera tryoni (Diptera: Tephritidae) for Quarantine Purposes. Ann. Entomol. Soc. Am. **105**, 434–446 (2012).

132. Fornoff, F., Dechmann, D. K. N. & Wikelski, M. Observation of movement and activity via radio-telemetry reveals diurnal behavior of the neotropical katydid Philophyllia Ingens (Orthoptera: Tettigoniidae). (2012).

133. Lorch, P. D., Sword, G. A., Gwynne, D. T. & Anderson, G. L. Radiotelemetry reveals differences in individual movement patterns between outbreak and non-outbreak Mormon cricket populations. Ecol. Entomol. **30**, 548–555 (2005).

134. Fermon, H., Waltert, M. & Mühlenberg, M. Movement and vertical stratification of fruit-feeding butterflies in a managed West African rainforest. J. Insect Conserv. **7**, 7–19 (2003).

135. Vlasanek, P., Sam, L. & Novotny, V. Dispersal of butterflies in a New Guinea rainforest: using mark–recapture methods in a large, homogeneous habitat. Ecol. Entomol. **38**, 560–569 (2013).

136. Lourenço, G. M., Dáttilo, W., Ribeiro, S. P. & Freitas, A. V. L. Biological Aspects and Movements of Neotropical Fruit-Feeding Butterflies. Neotrop. Entomol. **51**, 43–53 (2022).

137. Ehrlich, P. R. Intrinsic Barriers to Dispersal in Checkerspot Butterfly. Science **134**, 108–109 (1961).

138. Edwards, C. A. & Arancon, N. Q. Earthworm Physiology. in Biology and Ecology of Earthworms (eds. Edwards, C. A. & Arancon, N. Q.) 33–54 (Springer US, New York, NY, 2022).

139. Petersen, H. & Luxton, M. A Comparative Analysis of Soil Fauna Populations and Their Role in Decomposition Processes. Oikos **39**, 288–388 (1982).

140. Bastardie, F., Capowiez, Y., Renault, P. & Cluzeau, D. A radio-labelled study of earthworm behaviour in artificial soil cores in term of ecological types. Biol. Fertil. Soils **41**, 320–327 (2005).

141. van den Hoogen, J. et al. Soil nematode abundance and functional group composition at a global scale. Nature **572**, 194–198 (2019).

142. Dusenbery, D. B. A simple animal can use a complex stimulus pattern to find a location: Nematode thermotaxis in soil. Biol. Cybern. **60**, 431–437 (1989).

143. Pline, M. & Dusenbery, D. B. Responses of plant-parasitic nematodeMeloidogyne incognita to carbon dioxide determined by video camera-computer tracking. J. Chem. Ecol. **13**, 873–888 (1987).

144. Bal, H. K. & Grewal, P. S. Lateral Dispersal and Foraging Behavior of Entomopathogenic Nematodes in the Absence and Presence of Mobile and Non-Mobile Hosts. PLoS One **10**, e0129887 (2015).

145. Bal, H. K., Taylor, R. A. J. & Grewal, P. S. Ambush foraging entomopathogenic nematodes employ 'sprinters' for long-distance dispersal in the absence of hosts. J. Parasitol. **100**, 422–432 (2014).

146. Pinkerton, J. N., Mojtahedi, H., Santo, G. S. & O'Bannon, J. H. Vertical Migration of Meloidogyne chitwoodi and M. hapla under Controlled Temperature. J. Nematol. **19**, 152–157 (1987).

147. Bal, H. K., Taylor, R. A. J. & Grewal, P. S. Lateral movement of the entomopathogenic nematode Heterorhabditis bacteriophora in soil under laboratory conditions. 2009 OARDC Annual Research Conference (2009).

148. Jabbour, R. & Barbercheck, M. E. Soil and habitat complexity effects on movement of the entomopathogenic nematode Steinernema carpocapsae in maize. Biol. Control **47**, 235–243 (2008).

149. Gillooly, J. F., Gomez, J. P. & Mavrodiev, E. V. A broad-scale comparison of aerobic activity levels in vertebrates: endotherms versus ectotherms. Proc. Biol. Sci. **284**, (2017).

150. Feldman, A., Sabath, N., Pyron, R. A., Mayrose, I. & Meiri, S. Body sizes and diversification rates

of lizards, snakes, amphisbaenians and the tuatara. Glob. Ecol. Biogeogr. **25**, 187–197 (2016).

151. SWOT Report, Vol 15 — The State of the World's Sea Turtles. The State of the World's Sea Turtles | SWOT https://www.seaturtlestatus.org/swot-report-vol-15.
152. Olson, D. M. et al. Terrestrial Ecoregions of the World: A New Map of Life on Earth: A new global map of terrestrial ecoregions provides an innovative tool for conserving biodiversity. Bioscience **51**, 933–938 (2001).
153. Kochová, P. & Tonar, Z. Structural and mechanical properties of gastropod connective and smooth muscle tissue. Exp. Mech. **54**, 791–803 (2014).
154. Pembury Smith, M. Q. R. & Ruxton, G. D. How fast is a snail's pace? The influences of size and substrate on gastropod speed of locomotion. J. Zool. (1987) **314**, 12–19 (2021).
155. Fleming, T. H. Bat Migration. in Encyclopedia of Animal Behavior 605–610 (Elsevier, 2019).
156. Calderón-Capote, M. C. et al. Foraging movements are density-independent among straw-coloured fruit bats. R. Soc. Open Sci. **7**, 200274 (2020).
157. Robinson, M. F. & Stebbings, R. E. Home range and habitat use by the serotine bat, Eptesicus serotinus, in England. J. Zool. (1987) **243**, 117–136 (1997).
158. Meyer, C. F. J., Weinbeer, M. & Kalko, E. K. V. Home-Range Size and Spacing Patterns of Macrophyllum macrophyllum (Phyllostomidae) Foraging over Water. J Mammal **86**, 587–598 (2005).
159. Trevelin, L. C., Silveira, M., Port-Carvalho, M., Homem, D. H. & Cruz-Neto, A. P. Use of space by frugivorous bats (Chiroptera: Phyllostomidae) in a restored Atlantic forest fragment in Brazil. For. Ecol. Manage. **291**, 136–143 (2013).
160. Monadjem, A., Reside, A., Cornut, J. & Perrin, M. R. Roost selection and home range of an African insectivorous bat Nycteris thebaica (Chiroptera, Nycteridae). Mammalia **73**, 353–359 (2009).
161. Murray, S. W. & Kurta, A. Nocturnal activity of the endangered Indiana bat (Myotis sodalis). J. Zool. (1987) **262**, 197–206 (2004).
162. Winter, Y., von Helversen, O., Norberg, U. M., Kunz, T. H. & Steffensen, J. F. Flight cost and economy of nectar-feeding in the bat Glossophaga Soricina (Phyllostomidae; Glossophaginae). (1993).
163. Kalko, E. K. V., Estrada Villegas, S., Schmidt, M., Wegmann, M. & Meyer, C. F. J. Flying high--assessing the use of the aerosphere by bats. Integr. Comp. Biol. **48**, 60–73 (2008).
164. O'Donnell, C. F. J. Home range and use of space by Chalinolobus tuberculatus, a temperate rainforest bat from New Zealand. Journal of Zoology **253**, 253–264 (2001).
165. Almenar, D., Aihartza, J., Goiti, U., Salsamendi, E. & Garin, I. Reproductive and age classes do not change spatial dynamics of foraging long-fingered bats (Myotis capaccinii). Eur. J. Wildl. Res. **57**, 929–937 (2011).
166. Dietz, M. & Kalko, E. K. V. Reproduction affects flight activity in female and male Daubenton's bats, Myotis daubentoni. Can. J. Zool. **85**, 653–664 (2007).
167. Krauel, J. J., McGuire, L. P. & Boyles, J. G. Testing traditional assumptions about regional migration in bats. Mamm. Res. **63**, 115–123 (2018).
168. Pandey, H. O. & Upadhyay, D. Chapter Three - Global livestock production systems: Classification, status, and future trends. in Emerging Issues in Climate Smart Livestock Production (eds. Mondal, S. & Singh, R. L.) 47–70 (Academic Press, 2022).

169. Kilgour, R. J. In pursuit of ‘normal’: A review of the behaviour of cattle at pasture. Appl. Anim. Behav. Sci. **138**, 1–11 (2012).

170. Bailey, D. W., Vanwagoner, H. C., Weinmeister, R. & Jensen, D. Comparison of low-moisture blocks and salt for manipulating grazing patterns of beef cows. J. Anim. Sci. **86**, 1271–1277 (2008).

171. J. D. Davis, M. J. Darr, H. Xin, J. D. Harmon & J. R. Russell. Development of a GPS herd activity and well-being kit (GPS HAWK) to monitor cattle behavior and the effect of sample interval on travel distance. Appl. Eng. Agric. **27**, 143–150 (2011).

172. Clark, P. E. et al. Effects of Wolf Presence on Daily Travel Distance of Range Cattle. Rangeland Ecol. Manage. **70**, 657–665 (2017).

173. Ekwem, D. et al. Livestock movement informs the risk of disease spread in traditional production systems in East Africa. Sci. Rep. **11**, 16375 (2021).

174. Maruyama, H. & Nihei, T. Grazing Behavior of Cows Measured by Handheld GPS and Bite Counter Collar : A Case of Fazenda Baía Bonita in South Pantanal, Brazil. Japanese Journal of Human Geography **59**, 30–43 (2007).

175. Fierro, L. C. & Bryant, F. C. Grazing activities and bioenergetics of sheep on native range in Southern Peru. Small Rumin. Res. **3**, 135–146 (1990).

176. Lin, L., Dickhoefer, U., Müller, K., Wurina & Susenbeth, A. Grazing behavior of sheep at different stocking rates in the Inner Mongolian steppe, China. Appl. Anim. Behav. Sci. **129**, 36–42 (2011).

177. Food and Agriculture Organization of the United Nations (FAO). FAOSTAT. http://www.fao.org/faostat/en/#data/QA (2021).

178. Schwartzkopf-Genswein, K. & Grandin, T. Cattle transport by road. in Livestock handling and transport 143–173 (CABI, UK, 2014).

179. Massot, A., Negre, F., Vinci, C. & Dinkel, T. Patterns of Livestock Transport in the EU and to Third Countries. https://trid.trb.org/View/1891251 (2021).

180. Research for ANIT Committee - Patterns of livestock transport in the EU and to third countries. https://www.europarl.europa.eu/thinktank/en/document/IPOL_IDA(2021)690883.

181. Andersen, K. H. et al. Characteristic Sizes of Life in the Oceans, from Bacteria to Whales. Ann. Rev. Mar. Sci. **8**, 217–241 (2016).

182. Kiørboe, T. How zooplankton feed: mechanisms, traits and trade-offs. Biol. Rev. Camb. Philos. Soc. **86**, 311–339 (2011).

183. Uye, S.-I. Length-weight relationships of important zooplankton from the Inland Sea of Japan. J. Oceanogr. Soc. Japan **38**, 149–158 (1982).

184. Andersen, K. H. Fish Ecology, Evolution, and Exploitation: A New Theoretical Synthesis. (Princeton University Press, 2019).

185. Longhurst, A. R. & Glen Harrison, W. Vertical nitrogen flux from the oceanic photic zone by diel migrant zooplankton and nekton. Deep Sea Res. A **35**, 881–889 (1988).

186. Bianchi, D. & Mislan, K. A. S. Global patterns of diel vertical migration times and velocities from acoustic data. Limnol. Oceanogr. **61**, 353–364 (2016).

187. Nachtigall, W. Hydromechanics and biology. Biophys. Struct. Mech. **8**, 1–22 (1981).

188. Videler, J. J. & He, P. Swimming in Marine Fish. in Behavior of Marine Fishes 3–24 (Wiley-Blackwell, Oxford, UK, 2010).

189. Bale, R., Hao, M., Bhalla, A. P. S. & Patankar, N. A. Energy efficiency and allometry of movement of swimming and flying animals. Proc. Natl. Acad. Sci. U. S. A. **111**, 7517–7521 (2014).

190. Sato, K. et al. Stroke frequency, but not swimming speed, is related to body size in free-ranging seabirds, pinnipeds and cetaceans. Proc. Biol. Sci. **274**, 471–477 (2007).

191. Peters, R. H., Demers, E., Koelle, M. & MacKenzie, B. R. The allometry of swimming speed and predation. SIL Proceedings, 1922-2010 **25**, 2316–2323 (1994).

192. Helfman, G. S. Fish behaviour by day, night and twilight. in The Behaviour of Teleost Fishes 366–387 (Springer US, Boston, MA, 1986).

193. Froese, R., Thorson, J. T. & Reyes, R. B., Jr. A Bayesian approach for estimating length-weight relationships in fishes. J. Appl. Ichthyol. **30**, 78–85 (2014).

194. Froese, R. Cube law, condition factor and weight-length relationships: history, meta-analysis and recommendations. J. Appl. Ichthyol. **22**, 241–253 (2006).

195. Christiansen, S., Langangen, Ø., Titelman, J., Vøllestad, L. A. & Kaartvedt, S. Three-dimensional swimming behavior and activity of a mesopelagic fish. Limnol. Oceanogr. **67**, 2677–2690 (2022).

196. Sobradillo, B., Christiansen, S., Røstad, A. & Kaartvedt, S. Individual daytime swimming of mesopelagic fishes in the world's warmest twilight zone. Deep Sea Res. Part I **190**, 103897 (2022).

197. Christiansen, S., Titelman, J. & Kaartvedt, S. Nighttime swimming behavior of a mesopelagic fish. Front. Mar. Sci. **6**, (2019).

198. Magnabosco, C. et al. The biomass and biodiversity of the continental subsurface. Nat. Geosci. **11**, 707–717 (2018).

199. Palma, V., Gutiérrez, M. S., Vargas, O., Parthasarathy, R. & Navarrete, P. Methods to Evaluate Bacterial Motility and Its Role in Bacterial-Host Interactions. Microorganisms **10**, (2022).

200. Jarrell, K. F. & McBride, M. J. The surprisingly diverse ways that prokaryotes move. Nat. Rev. Microbiol. **6**, 466–476 (2008).

201. Bhattacharjee, T. & Datta, S. S. Confinement and activity regulate bacterial motion in porous media. Soft Matter **15**, 9920–9930 (2019).

202. Milo, R. & Phillips, R. Cell Biology by the Numbers. (CRC Press, Boca Raton, FL, 2015).

203. White, P. S., Morran, L. & de Roode, J. Phoresy. Curr. Biol. **27**, R578–R580 (2017).

204. Teske, P. R., Sandoval-Castillo, J., van Sebille, E., Waters, J. & Beheregaray, L. B. Oceanography promotes self-recruitment in a planktonic larval disperser. Sci. Rep. **6**, 34205 (2016).

205. Binks, R. M. et al. Habitat discontinuities form strong barriers to gene flow among mangrove populations, despite the capacity for long-distance dispersal. Divers. Distrib. **25**, 298–309 (2019).

206. In Brief to the State of World Fisheries and Aquaculture 2024. https://doi.org/10.4060/cd0690en (2024) doi:10.4060/cd0690en.

207. Sardain, A., Sardain, E. & Leung, B. Global forecasts of shipping traffic and biological invasions to 2050. Nature Sustainability **2**, 274–282 (2019).

208. Saebi, M. et al. Network analysis of ballast-mediated species transfer reveals important introduction and dispersal patterns in the Arctic. Sci. Rep. **10**, 19558 (2020).

209. Bai, Y. & Jin, W.-L. Green Ship Concepts. in Marine Structural Design 39–47 (Elsevier, 2016).

210. Lincoln P. Brower, Linda S. Fink, Peter Walford. Fueling the fall migration of the monarch butterfly. Integr. Comp. Biol. **46**, 1123–1142 (2006).

211. Egevang, C. et al. Tracking of Arctic terns Sterna paradisaea reveals longest animal migration. Proc. Natl. Acad. Sci. U. S. A. **107**, 2078–2081 (2010).

212. BirdLife International (BirdLife International). IUCN Red List of Threatened Species: Sterna paradisaea. IUCN Red List of Threatened Species (2018).

213. Droghini, A. & Boutin, S. The calm during the storm: Snowfall events decrease the movement rates of grey wolves (Canis lupus). PLoS One **13**, e0205742 (2018).

214. Kusak, J., Skrbinšek, A. M. & Huber, D. Home ranges, movements, and activity of wolves (Canis lupus) in the Dalmatian part of Dinarids, Croatia. Eur. J. Wildl. Res. **51**, 254–262 (2005).

215. Torney, C. J., Hopcraft, J. G. C., Morrison, T. A., Couzin, I. D. & Levin, S. A. From single steps to mass migration: the problem of scale in the movement ecology of the Serengeti wildebeest. Philos. Trans. R. Soc. Lond. B Biol. Sci. **373**, (2018).

216. Grange, S. et al. What limits the Serengeti zebra population? Oecologia **140**, 523–532 (2004).

217. IUCN. Equus quagga: King, S.R.B. & Moehlman, P.D. IUCN Red List of Threatened Species IUCN https://doi.org/10.2305/iucn.uk.2016-2.rlts.t41013a45172424.en (2016).

218. IUCN. Eudorcas thomsonii: IUCN SSC Antelope Specialist Group. IUCN Red List of Threatened Species IUCN https://doi.org/10.2305/iucn.uk.2018-2.rlts.t8982a172360006.en (2018).

219. IUCN. Nanger granti: IUCN SSC Antelope Specialist Group. IUCN Red List of Threatened Species IUCN https://doi.org/10.2305/iucn.uk.2016-2.rlts.t8971a50186774.en (2016).

220. Rizzo, L. Y. & Schulte, D. A review of humpback whales' migration patterns worldwide and their consequences to gene flow. J. Mar. Biol. Assoc. U. K. **89**, 995–1002 (2009).

221. Fisheries, N. Humpback whale. NOAA https://www.fisheries.noaa.gov/species/humpback-whale (2024).

222. Cooke, J. IUCN Red List of Threatened Species: Megaptera novaeangliae. IUCN Red List of Threatened Species (2018).

223. Klevjer, T. A. et al. Large scale patterns in vertical distribution and behaviour of mesopelagic scattering layers. Sci. Rep. **6**, 19873 (2016).

224. El Hanandeh, A. Quantifying the carbon footprint of religious tourism: the case of Hajj. J. Clean. Prod. **52**, 53–60 (2013).

225. South Pole (South Pole Carbon Asset Management Ltd.), S., S. & ASTAD. Greenhouse Gas Accounting Report. FIFA World Cup 2022™. (FIFA (Fédération Internationale de Football Association), 2021).

226. IATA. World Air Transport Statistics 2019.

227. Lyons, S. K., Smith, F. A. & Brown, J. H. Of mice, mastodons, and men: Human mediated extinctions on four continents. *Evol Ecol Res* **6**, 339–358 (2004).

228. Koch, P. L. & Barnosky, A. D. Late Quaternary extinctions: State of the debate. *Annu. Rev. Ecol. Evol. Syst.* **37**, 215–250 (2006).

229. Silva, M. & Downing, J. A. The allometric scaling of density and body mass: A nonlinear relationship for terrestrial mammals. *Am. Nat.* **145**, 704–727 (1995).

230. Letcher, A. J. & Harvey, P. H. Variation in geographical range size among mammals of the palearctic. *Am. Nat.* **144**, 30–42 (1994).

231. Greenspoon, L. *et al.* Reply to Santini et al.: Total population reports are necessary for global

biomass estimation of wild mammals. *Proc. Natl. Acad. Sci. U. S. A.* **121**, e2316314121 (2024).

232. ITF. ITF Transport Statistics. Passenger transport OECD. https://doi.org/10.1787/trsprt-data-en, (accessed 04 Mar 2024).
