# Supplementary material for: Human biomass movement exceeds the biomass movement of all land animals combined
Source: Nat Ecol Evol. 2025 Oct 27;9(12):2259–64. doi: 10.1038/s41559-025-02863-9 (PMC12680537; doi:10.1038/s41559-025-02863-9)
Supplement: Supplementary file 2 — Reporting Summary [file 41559_2025_2863_MOESM2_ESM.pdf]

nature portfolio

Corresponding author(s): Ron Milo

Last updated by author(s): Aug 4, 2025

Reporting Summary

Nature Portfolio wishes to improve the reproducibility of the work that we publish. This form provides structure for consistency and transparency in reporting. For further information on Nature Portfolio policies, see our Editorial Policies and the Editorial Policy Checklist.

Statistics

For all statistical analyses, confirm that the following items are present in the figure legend, table legend, main text, or Methods section.

n/a	Confirmed	
☐	☒	The exact sample size (*n*) for each experimental group/condition, given as a discrete number and unit of measurement
☒	☐	A statement on whether measurements were taken from distinct samples or whether the same sample was measured repeatedly
☒	☐	The statistical test(s) used AND whether they are one- or two-sided<br>*Only common tests should be described solely by name; describe more complex techniques in the Methods section.*
☒	☐	A description of all covariates tested
☐	☒	A description of any assumptions or corrections, such as tests of normality and adjustment for multiple comparisons
☐	☒	A full description of the statistical parameters including central tendency (e.g. means) or other basic estimates (e.g. regression coefficient) AND variation (e.g. standard deviation) or associated estimates of uncertainty (e.g. confidence intervals)
☒	☐	For null hypothesis testing, the test statistic (e.g. *F*, *t*, *r*) with confidence intervals, effect sizes, degrees of freedom and *P* value noted<br>*Give P values as exact values whenever suitable.*
☒	☐	For Bayesian analysis, information on the choice of priors and Markov chain Monte Carlo settings
☒	☐	For hierarchical and complex designs, identification of the appropriate level for tests and full reporting of outcomes
☒	☐	Estimates of effect sizes (e.g. Cohen's *d*, Pearson's *r*), indicating how they were calculated

Our web collection on statistics for biologists contains articles on many of the points above.

Software and code

Policy information about availability of computer code

Data collection	*Provide a description of all commercial, open source and custom code used to collect the data in this study, specifying the version used OR state that no software was used.*
Data analysis	We have used JupyterLab version 4.0.11 with Python version 3.12.4 , and Microsoft Excel version 16.77.1 to analyze data. The data and code are publicly available at https://doi.org/10.5281/zenodo.16731771

For manuscripts utilizing custom algorithms or software that are central to the research but not yet described in published literature, software must be made available to editors and reviewers. We strongly encourage code deposition in a community repository (e.g. GitHub). See the Nature Portfolio guidelines for submitting code & software for further information.

Data

Policy information about availability of data

All manuscripts must include a data availability statement. This statement should provide the following information, where applicable:

- Accession codes, unique identifiers, or web links for publicly available datasets
- A description of any restrictions on data availability
- For clinical datasets or third party data, please ensure that the statement adheres to our policy

All data generated and used to produce the results described in this study is publicly available at https://doi.org/10.5281/zenodo.16731771.
We have also used data from Movebank database which can be downloaded via its API with the provided code.

Research involving human participants, their data, or biological material

Policy information about studies with human participants or human data. See also policy information about sex, gender (identity/presentation), and sexual orientation and race, ethnicity and racism.

Reporting on sex and gender	The data on human mobility was collected by various organizations independently of this work, as described in the main text and supplementary information. It typically did not include information regarding sex or gender. Our results regarding human biomass movement apply to both sexes.
Reporting on race, ethnicity, or other socially relevant groupings	We have used the grouping of countries into standard economic income groups, as developed by the World Bank. This categorization was part of our biomass movement extrapolation procedure, as countries under the same economic income group share relevant human mobility characteristics. Further details are found in the supplementary information.
Population characteristics	*Describe the covariate-relevant population characteristics of the human research participants (e.g. age, genotypic information, past and current diagnosis and treatment categories). If you filled out the behavioural & social sciences study design questions and have nothing to add here, write "See above."*
Recruitment	*Describe how participants were recruited. Outline any potential self-selection bias or other biases that may be present and how these are likely to impact results.*
Ethics oversight	*Identify the organization(s) that approved the study protocol.*

Note that full information on the approval of the study protocol must also be provided in the manuscript.

Field-specific reporting

Please select the one below that is the best fit for your research. If you are not sure, read the appropriate sections before making your selection.

☐ Life sciences ☐ Behavioural & social sciences ☒ Ecological, evolutionary & environmental sciences

For a reference copy of the document with all sections, see nature.com/documents/nr-reporting-summary-flat.pdf

Ecological, evolutionary & environmental sciences study design

All studies must disclose on these points even when the disclosure is negative.

Study description	We synthesized hundreds of studies and data sources and utilized diverse approaches to coherently evaluate the biomass movement of all animals and humans on Earth.
Research sample	Our data is based on the published literature, the Movebank database, the AVONET database, BirdLife International, the IUCN, and the referenced agencies, governments, and organizations. It includes information on animal biomass, mobility, and other characteristics.
Sampling strategy	We used all the relevant data that we could find to form our samples.
Data collection	Data was collected from published literature and databases by YR, DW, DV, GBS, LG, BH. We did not collect original data (e.g. we did not track animals ourselves)
Timing and spatial scale	Our data spans across the world and multiple years, seasons, and times of the day. We aimed at collecting data that can be averaged on an annual basis.
Data exclusions	We excluded bird movement data we suspected to be erroneous or could not be used to infer typical daily movement patterns, as detailed in the supplementary information. The exclusion criteria were pre-established.
Reproducibility	Our study does not include experiments.
Randomization	We grouped our animal data by taxonomy and trait and tested the sensitivity of the results to the various groupings, as discussed in the manuscript. The effects of the related potential biases is incorporated into our uncertainty analysis. We also estimate upper bounds as an alternative way to account for potential biases in the data and data scarcity.
Blinding	Blinding was irrelevant to our study during data acquisition, as the data was preexisting, objective, and we collected all the available data we could find. Blinding of the animal taxonomy was not possible during analysis, as the analysis was partly based on taxonomy. However, the analysis was based on objective measures and established protocols when possible, mitigating potential biases.

Did the study involve field work? ☐ Yes ☒ No

Reporting for specific materials, systems and methods

We require information from authors about some types of materials, experimental systems and methods used in many studies. Here, indicate whether each material, system or method listed is relevant to your study. If you are not sure if a list item applies to your research, read the appropriate section before selecting a response.

Materials & experimental systems

n/a	Involved in the study
☒	☐ Antibodies
☒	☐ Eukaryotic cell lines
☒	☐ Palaeontology and archaeology
☒	☐ Animals and other organisms
☒	☐ Clinical data
☒	☐ Dual use research of concern
☒	☐ Plants

Methods

n/a	Involved in the study
☒	☐ ChIP-seq
☒	☐ Flow cytometry
☒	☐ MRI-based neuroimaging

Plants

Seed stocks	*Report on the source of all seed stocks or other plant material used. If applicable, state the seed stock centre and catalogue number. If plant specimens were collected from the field, describe the collection location, date and sampling procedures.*
Novel plant genotypes	*Describe the methods by which all novel plant genotypes were produced. This includes those generated by transgenic approaches, gene editing, chemical/radiation-based mutagenesis and hybridization. For transgenic lines, describe the transformation method, the number of independent lines analyzed and the generation upon which experiments were performed. For gene-edited lines, describe the editor used, the endogenous sequence targeted for editing, the targeting guide RNA sequence (if applicable) and how the editor was applied.*
Authentication	*Describe any authentication procedures for each seed stock used or novel genotype generated. Describe any experiments used to assess the effect of a mutation and, where applicable, how potential secondary effects (e.g. second site T-DNA insertions, mosiacism, off-target gene editing) were examined.*
